# Surveillance for Violent Deaths — National Violent Death Reporting System, 34 States, Four California Counties, the District of Columbia, and Puerto Rico, 2017

**DOI:** 10.15585/mmwr.ss6908a1

**Published:** 2020-12-04

**Authors:** Emiko Petrosky, Allison Ertl, Kameron J. Sheats, Rebecca Wilson, Carter J. Betz, Janet M. Blair

**Affiliations:** 1 Division of Violence Prevention, National Center for Injury Prevention and Control, CDC

## Abstract

**Problem/Condition:**

In 2017, approximately 67,000 persons died of violence-related injuries in the United States. This report summarizes data from CDC’s National Violent Death Reporting System (NVDRS) on violent deaths that occurred in 34 states, four California counties, the District of Columbia, and Puerto Rico in 2017. Results are reported by sex, age group, race/ethnicity, method of injury, type of location where the injury occurred, circumstances of injury, and other selected characteristics.

**Period Covered:**

2017.

**Description of System:**

NVDRS collects data regarding violent deaths obtained from death certificates, coroner and medical examiner reports, and law enforcement reports. This report includes data collected for violent deaths that occurred in 2017. Data were collected from 34 states (Alaska, Arizona, Colorado, Connecticut, Delaware, Georgia, Illinois, Indiana, Iowa, Kansas, Kentucky, Maine, Maryland, Massachusetts, Michigan, Minnesota, Nevada, New Hampshire, New Jersey, New Mexico, New York, North Carolina, Ohio, Oklahoma, Oregon, Pennsylvania, Rhode Island, South Carolina, Utah, Vermont, Virginia, Washington, West Virginia, and Wisconsin), four California counties (Los Angeles, Sacramento, Shasta, and Siskiyou), the District of Columbia, and Puerto Rico. NVDRS collates information for each death and links deaths that are related (e.g., multiple homicides, homicide followed by suicide, or multiple suicides) into a single incident.

**Results:**

For 2017, NVDRS collected information on 45,141 fatal incidents involving 46,389 deaths that occurred in 34 states, four California counties, and the District of Columbia; in addition, information was collected on 961 fatal incidents involving 1,027 deaths in Puerto Rico. Data for Puerto Rico were analyzed separately. Of the 46,389 deaths in the 34 states, four California counties, and District of Columbia, the majority (63.5%) were suicides, followed by homicides (24.9%), deaths of undetermined intent (9.7%), legal intervention deaths (1.4%) (i.e., deaths caused by law enforcement and other persons with legal authority to use deadly force acting in the line of duty, excluding legal executions), and unintentional firearm deaths (<1.0%). (The term “legal intervention” is a classification incorporated into the *International Classification of Diseases, Tenth Revision,* and does not denote the lawfulness or legality of the circumstances surrounding a death caused by law enforcement.) Demographic patterns and circumstances varied by manner of death. The suicide rate was higher among males than among females and was highest among adults aged 45–64 years and ≥85 years and non-Hispanic American Indians/Alaska Natives and non-Hispanic Whites. The most common method of injury for suicide was a firearm among males and poisoning among females. Suicide was most often preceded by a mental health, intimate partner, or physical health problem or a recent or impending crisis during the previous or upcoming 2 weeks. The homicide rate was highest among persons aged 20–24 years and was higher among males than females. Non-Hispanic Black males had the highest homicide rate of any racial/ethnic group. The most common method of injury for homicide was a firearm. When the relationship between a homicide victim and a suspect was known, the suspect was most frequently an acquaintance or friend for male victims and a current or former intimate partner for female victims. Homicide most often was precipitated by an argument or conflict, occurred in conjunction with another crime, or, for female victims, was related to intimate partner violence. Among intimate partner violence–related homicides, the largest proportion occurred among adults aged 35–54 years, and the most common method of injury was a firearm. When the relationship between an intimate partner violence–related homicide victim and a suspect was known, most female victims were killed by a current or former intimate partner, whereas approximately half of male victims were killed by a suspect who was not their intimate partner. Almost all legal intervention deaths were among males, and the legal intervention death rate was highest among men aged 25–29 years. Non-Hispanic American Indian/Alaska Native males had the highest legal intervention death rate, followed by non-Hispanic Black males. A firearm was used in the majority of legal intervention deaths. When a specific type of crime was known to have precipitated a legal intervention death, the type of crime was most frequently assault/homicide. The most frequent circumstances for legal intervention deaths were reported use of a weapon by the victim in the incident and a mental health or substance use problem (other than alcohol use). Unintentional firearm deaths more frequently occurred among males, non-Hispanic Whites, and persons aged 15–24 years. These deaths most often occurred while the shooter was playing with a firearm and most frequently were precipitated by a person unintentionally pulling the trigger or mistakenly thinking the firearm was unloaded. The rate of death when the manner was of undetermined intent was highest among males, particularly among non-Hispanic Black and non-Hispanic American Indian/Alaska Native males, and persons aged 30–34 years. Poisoning was the most common method of injury in deaths of undetermined intent, and opioids were detected in nearly 80% of decedents tested for those substances.

**Interpretation:**

This report provides a detailed summary of data from NVDRS on violent deaths that occurred in 2017. The suicide rate was highest among non-Hispanic American Indian/Alaska Native and non-Hispanic White males, whereas the homicide rate was highest among non-Hispanic Black males. Intimate partner violence precipitated a large proportion of homicides for females. Mental health problems, intimate partner problems, interpersonal conflicts, and acute life stressors were primary circumstances for multiple types of violent death.

**Public Health Action:**

NVDRS data are used to monitor the occurrence of violence-related fatal injuries and assist public health authorities in developing, implementing, and evaluating programs and policies to reduce and prevent violent deaths. For example, South Carolina VDRS and Colorado VDRS are using their data to support suicide prevention programs through systems change and the *Zero Suicide* framework. North Carolina VDRS and Kentucky VDRS data were used to examine intimate partner violence–related deaths beyond homicides to inform prevention efforts. Findings from these studies suggest that intimate partner violence might also contribute to other manners of violent death, such as suicide, and preventing intimate partner violence might reduce the overall number of violent deaths. In 2019, NVDRS expanded data collection to include all 50 states, the District of Columbia, and Puerto Rico, providing more comprehensive and actionable violent death information for public health efforts to reduce violent deaths.

## Introduction

In 2017, violence-related injuries led to approximately 67,000 deaths in the United States (*1*). Suicide was the 10th leading cause of death overall in the United States and disproportionately affected young and middle-aged populations. By age group, suicide was the second leading cause of death for persons aged 10–34 years and the fourth leading cause of death for persons aged 35–54 years. During 2017, non-Hispanic American Indian/Alaska Native and non-Hispanic White males were disproportionately affected by suicide.

In 2017, homicide was the 16th leading cause of death overall in the United States but disproportionately affected young persons (*1*). Homicide was among the five leading causes of death for children aged 1–14 years and was the third leading cause of death for persons aged 15–34 years. Young non-Hispanic Black males also were disproportionately affected by homicide. Homicide was the leading cause of death for non-Hispanic Black males aged 15–34 years and the second leading cause of death for non-Hispanic Black males aged 1–14 years.

Public health authorities require accurate, timely, and complete surveillance data to better understand and ultimately prevent the occurrence of violent deaths in the United States (*2*). In 2000, in response to an Institute of Medicine* report noting the need for a national fatal intentional injury surveillance system (*3*), CDC began planning to implement the National Violent Death Reporting System (NVDRS) (*2*). The goals of NVDRS are to

- collect and analyze timely, quality data for monitoring the magnitude and characteristics of violent deaths at national, state, and local levels;
- ensure data are disseminated routinely and expeditiously to public health officials, law enforcement officials, policymakers, and the public;
- ensure data are used to develop, implement, and evaluate programs and strategies that are intended to reduce and prevent violent deaths and injuries at national, state, and local levels; and
- build and strengthen partnerships among organizations and communities at national, state, and local levels to ensure that data are collected and used to reduce and prevent violent deaths and injuries.

NVDRS is a state-based active surveillance system that collects data on the characteristics and circumstances associated with violence-related deaths in participating states, districts, and territories (*2*). Deaths collected by NVDRS include suicides, homicides, legal intervention deaths (i.e., deaths caused by law enforcement acting in the line of duty and other persons with legal authority to use deadly force, excluding legal executions), unintentional firearm deaths, and deaths of undetermined intent that might have been due to violence.^†^ The term “legal intervention” is a classification incorporated into the *International Classification of Diseases, Tenth Revision,* (*ICD-10*) (*4*) and does not denote the lawfulness or legality of the circumstances surrounding a death caused by law enforcement.

Before implementation of NVDRS, single data sources (e.g., death certificates) provided only limited information and few circumstances from which to understand patterns of violent deaths. NVDRS filled this surveillance gap by providing more detailed information. NVDRS is the first system to 1) provide detailed information on circumstances precipitating violent deaths, 2) link multiple source documents so that each incident can contribute to the study of patterns of violent deaths, and 3) link multiple deaths that are related to one another (e.g., multiple homicides, suicide pacts, or homicide followed by suicide of the suspect).

NVDRS data collection began in 2003 with six participating states (Maryland, Massachusetts, New Jersey, Oregon, South Carolina, and Virginia) (Figure). Seven states (Alaska, Colorado, Georgia, North Carolina, Oklahoma, Rhode Island, and Wisconsin) began data collection in 2004, three (Kentucky, New Mexico, and Utah) in 2005, two (Ohio and Michigan) in 2010, and 14 (Arizona, Connecticut, Hawaii, Illinois, Indiana, Iowa, Kansas, Maine, Minnesota, New Hampshire, New York, Pennsylvania, Vermont, and Washington) in 2015. In 2017, eight additional states began data collection (Alabama, California, Delaware, Louisiana, Missouri, Nebraska, Nevada, and West Virginia), along with the District of Columbia and Puerto Rico.^§^ NVDRS received funding in 2018 for a nationwide expansion that included the remaining 10 states (Arkansas, Florida, Idaho, Mississippi, Montana, North Dakota, South Dakota, Tennessee, Texas, and Wyoming), which began data collection in 2019. CDC now provides NVDRS funding to all 50 states, the District of Columbia, and Puerto Rico. NVDRS data are updated annually and are available to the public through CDC’s Web-based Injury Statistics Query and Reporting System (WISQARS)^¶^ at https://www.cdc.gov/injury/wisqars/nvdrs.html. Case-level NVDRS data are available to interested researchers who meet eligibility requirements via access to the NVDRS Restricted Access Database (https://www.cdc.gov/ViolencePrevention/NVDRS/RAD.html).

**FIGURE:**
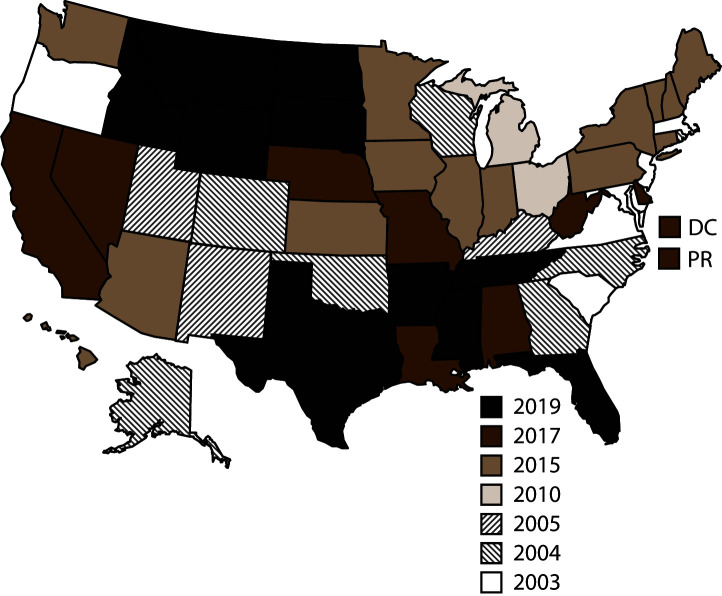
States participating in the National Violent Death Reporting System, by year of initial data collection* — United States and Puerto Rico, 2003–2020 **Abbreviations:** DC = District of Columbia; PR = Puerto Rico. * California began collecting data for a subset of violent deaths in 2005 but ended data collection in 2009. In 2017, California collected data from death certificates for all violent deaths in the state. Data for violent deaths that occurred in four counties (Los Angeles, Sacramento, Shasta, and Siskiyou) included information from all three required sources (i.e., death certificates, coroner or medical examiner reports, and law enforcement reports). Michigan collected data for a subset of violent deaths during 2010–2013 and collected statewide data beginning in 2014. In 2016, Illinois, Pennsylvania, and Washington began collecting data on violent deaths for a subset of counties that represented at least 80% of all violent deaths in their state, or in counties where at least 1,800 violent deaths occurred. Beginning in 2019, all 50 U.S. states, the District of Columbia, and Puerto Rico were participating in the system.

This report summarizes NVDRS data on violent deaths that occurred in 34 states, four California counties, the District of Columbia, and Puerto Rico in 2017 and highlights the proportion of homicides precipitated by intimate partner violence. Homicide is a leading cause of death among males and females, particularly among young persons (*1*), and approximately half of female homicide victims are killed by a current or former male intimate partner (*5*). The high proportion of intimate partner violence–related homicides, particularly among females, warrants a more comprehensive understanding of the characteristics and circumstances of these deaths to support intimate partner violence prevention efforts.

## Methods

NVDRS compiles information from three required data sources: death certificates, coroner and medical examiner reports, and law enforcement reports (*2*). Certain participating Violent Death Reporting System (VDRS) programs might also collect information from secondary sources (e.g., child fatality review team data, Federal Bureau of Investigation Supplementary Homicide Reports, and crime laboratory data). NVDRS combines information for each death and links deaths that are related (e.g., multiple homicides, homicide followed by suicide, or multiple suicides) into a single incident. The ability to analyze linked data can provide a more comprehensive understanding of violent deaths. Participating VDRS programs use vital statistics death certificate files or coroner or medical examiner reports to identify violent deaths meeting the NVDRS case definition. Each VDRS program reports violent deaths of residents that occurred within the state, district, or territory and those of nonresidents for whom a fatal injury occurred within the state, district, or territory (i.e., occurrent deaths). When a violent death is identified, NVDRS data abstractors link source documents, link deaths within each incident, code data elements, and write brief narratives of the incident.

In NVDRS, a violent death is defined as a death resulting from the intentional use of physical force or power, threatened or actual, against oneself, another person, or a group or community (*6*). NVDRS collects information on five manners of death: 1) suicide, 2) homicide, 3) legal intervention death, 4) unintentional firearm death, and 5) death of undetermined intent that might have been due to violence (see Manner of Death). Information also is collected about unintentional firearm deaths (i.e., a death resulting from a penetrating injury or gunshot wound from a weapon that uses a powder charge to fire a projectile when a preponderance of evidence indicates that the shooting was not intentionally directed at the victim) and deaths of undetermined intent that might have been due to violence (i.e., a death that results from the use of force or power against oneself or another person for which the evidence indicating one manner of death is no more compelling than evidence indicating another). NVDRS cases are coded on the basis of *ICD-10* (*4*) or the manner of death assigned by a coroner, medical examiner, or law enforcement officer. Cases are included if they are assigned *ICD-10* codes (Box 1) or a manner of death specified in at least one of the three primary data sources consistent with NVDRS case definitions.

BOX 1 ***International Classification of Diseases, Tenth Revision* (*ICD-10*), codes used in the National Violent Death Reporting System**

**Table:** 

Manner of death	Death ≤1 year after injury	Death >1 year after injury	Death any time after injury
Intentional self-harm (suicide)	X60–X84	Y87.0	U03 (attributable to terrorism)
Assault (homicide)	X85–X99, Y00–Y09	Y87.1	U01, U02 (attributable to terrorism)
Event of undetermined intent	Y10–Y34	Y87.2, Y89.9	Not applicable
Unintentional exposure to inanimate mechanical forces (firearms)	W32–W34	Y86	Not applicable
Legal intervention (excluding executions, Y35.5)	Y35.0–Y35.4, Y35.6, Y35.7	Y89.0	Not applicable

NVDRS is an incident-based system, and all decedents associated with a given incident are grouped in one record. Decisions about whether two or more deaths are related and belong to the same incident are made on the basis of the timing of the injuries rather than on the timing of the deaths. Deaths resulting from injuries that are clearly linked by source documents and occur within 24 hours of each other (see Manner of Death) would be considered part of the same incident. Examples of an incident include 1) a single isolated violent death, 2) two or more related homicides (including legal intervention deaths) when the fatal injuries were inflicted <24 hours apart, 3) two or more related suicides or deaths of undetermined intent when the fatal injuries were inflicted <24 hours apart, and 4) a homicide followed by a suicide when both fatal injuries were inflicted <24 hours apart (*7*).

Information collected from each data source is entered into the NVDRS web-based data entry system (*2*). This system streamlines data abstraction by allowing abstractors to enter data from multiple sources into the same incident record. Internal validation checks, hover-over features that define selected fields, and other quality control measures are included. Primacy rules and hierarchal algorithms related to the source documents occur at the local level. CDC provides access to the web-based system to each VDRS program. Program personnel are provided ongoing coding training to learn and adhere to CDC guidance regarding coding of all variables and technical assistance to help increase data quality. Data are transmitted continuously via the web to a CDC-based server. Information abstracted into the system is deidentified at the local VDRS program level.

### Manner of Death

A manner (i.e., intent) of death for each decedent is assigned by a trained abstractor who integrates information from all source documents. The abstractor-assigned manner of death must agree with at least one required data source; typically, all source documents are consistent regarding the manner of death. When a discrepancy exists, the abstractor must assign a manner of death on the basis of a preponderance of evidence in the source documents; however, such occurrences are rare (*7*). For example, if two sources report a death as a suicide and a third reports it as a death of undetermined intent, the death is coded as a suicide.

NVDRS data are categorized into five abstractor-assigned manners of death: 1) suicide, 2) homicide, 3) legal intervention death, 4) unintentional firearm death, and 5) death of undetermined intent. The case definitions for each manner of death are described as follows:

- **Suicide.** A suicide is a death among persons aged ≥10 years resulting from the use of force against oneself when a preponderance of evidence indicates that the use of force was intentional. This category also includes the following scenarios: 1) deaths of persons who intended only to injure rather than kill themselves; 2) persons who initially intended to kill themselves, changed their minds, but died as a result of the act; 3) deaths associated with risk-taking behavior without clear intent to inflict fatal self-injury but associated with high risk for death (e.g., participating in Russian roulette); 4) suicides that occurred while under the influence of substances or drugs, taken voluntarily; 5) suicides among decedents with mental illnesses that affected their thinking, feelings, or mood (e.g., while experiencing an acute episode of mental illness); and 6) suicides involving another person who provided only passive assistance to the decedent (e.g., supplying the means or information needed to complete the act). This category does not include deaths caused by chronic or acute substance use without the intent to die, deaths attributed to autoerotic behavior (e.g., self-strangulation during sexual activity), or assisted suicides (legal or nonlegal). Corresponding *ICD-10* codes included in NVDRS are X60–X84, Y87.0, and U03 (Box 1).
- **Homicide.** A homicide is a death resulting from the use of physical force or power, threatened or actual, against another person, group, or community when a preponderance of evidence indicates that the use of force was intentional. Two special scenarios that CDC’s National Center for Health Statistics (NCHS) regards as homicides are included in the NVDRS case definition: 1) arson with no specified intent to injure someone and 2) a stabbing with intent unspecified. This category also includes the following scenarios: 1) deaths when the suspect intended to only injure rather than kill the victim, 2) deaths resulting from heart attack induced when the suspect used force or power against the victim, 3) deaths that occurred when a person killed an attacker in self-defense, 4) deaths resulting from a weapon that discharged unintentionally while being used to control or frighten a victim, 5) deaths attributed to child abuse without intent being specified, 6) deaths attributed to an intentional act of neglect by one person against another, 7) deaths of live-born infants that resulted from a direct injury due to violence sustained before birth, and 8) deaths identified as justifiable homicide when the person committing homicide was not a law enforcement officer. This category excludes vehicular homicide without intent to injure, unintentional poisoning deaths due to illicit or prescription drug overdose even when the person who provided drugs was charged with homicide, unintentional firearm deaths (a separate category in NVDRS), combat deaths or acts of war, deaths of unborn fetuses, and deaths of infants that resulted indirectly from violence sustained by the mother before birth (e.g., death from prematurity following premature labor brought on by violence). Corresponding *ICD-10* codes included in NVDRS are X85–X99, Y00–Y09, Y87.1, and U01–U02 (Box 1).
- **Legal intervention.** A death from legal intervention is a death in which a person is killed or died as a result of injuries inflicted by a law enforcement officer or other peace officer (i.e., a person with specified legal authority to use deadly force), including military police, while acting in the line of duty. The term “legal intervention” is a classification from *ICD-10* (Y-35.0) and does not denote the lawfulness or legality of the circumstances surrounding a death caused by law enforcement. Legal intervention deaths also include a small subset of cases in which force was applied without clear lethal intent (e.g., during restraint or when applying force with a typically nondeadly weapon, such as a Taser), or in which the death occurred while the person was fleeing capture. This category excludes legal executions. Corresponding *ICD-10* codes included in NVDRS are Y35.0–Y35.4, Y35.6, Y35.7, and Y89.0 (Box 1).
- **Unintentional firearm.** An unintentional firearm death is a death resulting from a penetrating injury or gunshot wound from a weapon that uses a powder charge to fire a projectile and for which a preponderance of evidence indicates that the shooting was not directed intentionally at the decedent. Examples include the following: 1) a person who died as a result of a celebratory firing that was not intended to frighten, control, or harm anyone; 2) a person who unintentionally shot themself when using a firearm to frighten, control, or harm another person; 3) a soldier who was shot during a field exercise but not in a combat situation; 4) a person who received a self-inflicted wound while playing with a firearm; 5) a person who mistakenly believed a gun was unloaded and shot another person; 6) a child aged <6 years who shot themself or another person; and 7) an infant who died after birth from an unintentional firearm injury that was sustained in utero. This category excludes injuries caused by unintentionally striking a person with the firearm (e.g., hitting a person on the head with the firearm rather than firing a projectile) and unintentional injuries from nonpowder guns (e.g., BB, pellet, or other compressed air–powered or gas-powered guns). Corresponding *ICD-10* codes included in NVDRS are W32–W34 and Y86 (Box 1).
- **Undetermined intent.** A death of undetermined intent in NVDRS is a death resulting from the use of force or power against oneself or another person for which the evidence indicating one manner of death is no more compelling than evidence indicating another. This category includes coroner or medical examiner rulings where records from data providers indicate that investigators did not find enough evidence to determine whether the injury was intentional (e.g., unclear whether a drug overdose was unintentional or a suicide). Corresponding *ICD-10* codes included in NVDRS are Y10–Y34, Y87.2, and Y89.9 (Box 1).

### Variables Analyzed

NVDRS collects approximately 600 unique variables for each death (Boxes 1, 2, and 3). The number of variables recorded for each incident depends on the content and completeness of the source documents. Variables in NVDRS include

BOX 2 **Methods used to inflict injury — National Violent Death Reporting System, 34 states, four California counties, the District of Columbia, and Puerto Rico, 2017**

- Firearm: method that uses a powder charge to fire a projectile from the weapon (excludes BB gun, pellet gun, compressed-air or gas-powered gun)
- Hanging/strangulation/suffocation (e.g., hanging by the neck, manual strangulation, or plastic bag over the head)
- Poisoning (e.g., fatal ingestion of a street drug, pharmaceutical, carbon monoxide, gas, rat poison, or insecticide)
- Sharp instrument (e.g., knife, razor, machete, or pointed instrument)
- Blunt instrument (e.g., club, bat, rock, or brick)
- Fall: being pushed or jumping
- Motor vehicle (e.g., car, bus, motorcycle, or other transport vehicle)
- Personal weapons (e.g., hands, fists, or feet)
- Drowning: inhalation of liquid (e.g., in bathtub, lake, or other source of water/liquid)
- Fire/burns: inhalation of smoke or the direct effects of fire or chemical burns
- Intentional neglect: starvation, lack of adequate supervision, or withholding of health care
- Other (single method): any method other than those already listed (e.g., electrocution, exposure to environment/weather, or explosives)
- Unknown: method not reported or not known

BOX 3 **Circumstances preceding fatal injury, by manner of death — National Violent Death Reporting System, 34 states, four California counties, the District of Columbia, and Puerto Rico, 2017**

**Suicide/Undetermined Intent**

- Intimate partner problem: decedent was experiencing problems with a current or former intimate partner.
- Suicide of family member or friend: decedent was distraught over, or reacting to, the suicide of a family member or friend.
- Other death of family member or friend: decedent was distraught over, or reacting to, the recent nonsuicide death of a family member or friend.
- Physical health problem: decedent was experiencing physical health problems (e.g., a recent cancer diagnosis or chronic pain).
- Job problem: decedent was either experiencing a problem at work or was having a problem with joblessness.
- Recent criminal legal problem: decedent was facing criminal legal problems (e.g., recent or impending arrest or upcoming criminal court date).
- Noncriminal legal problem: decedent was facing civil legal problems (e.g., a child custody or civil lawsuit).
- Financial problem: decedent was experiencing financial problems (e.g., bankruptcy, overwhelming debt, or foreclosure of a home or business).
- Eviction or loss of home: decedent was experiencing a recent or impending eviction or other loss of housing, or the threat of eviction or loss of housing.
- School problem: decedent was experiencing a problem related to school (e.g., poor grades, bullying, social exclusion at school, or performance pressures).
- Traumatic anniversary: the incident occurred on or near the anniversary of a traumatic event in the decedent’s life.
- Exposure to disaster: decedent was exposed to a disaster (e.g., earthquake or bombing).
- Left a suicide note: decedent left a note, e-mail message, video, or other communication indicating intent to die by suicide.
- Disclosed suicidal intent: decedent had recently expressed suicidal feelings to another person with time for that person to intervene.
- Disclosed intent to whom: type of person (e.g., family member or current or former intimate partner) to whom the decedent recently disclosed suicidal thoughts or plans.
- History of suicidal thoughts or plans: decedent had previously expressed suicidal thoughts or plans.
- History of suicide attempt: decedent had previously attempted suicide before the fatal incident.

**Homicide/Legal Intervention**

- Jealousy (lovers’ triangle): jealousy or distress over an intimate partner’s relationship or suspected relationship with another person.
- Stalking: pattern of unwanted harassing or threatening tactics by either the decedent or suspect.
- Prostitution: prostitution or related activity that includes prostitutes, pimps, clients, or others involved in such activity.
- Drug involvement: drug dealing, drug trade, or illicit drug use.
- Brawl: mutual physical fight involving three or more persons.
- Mercy killing: decedent wished to die because of a terminal or hopeless disease or condition, and documentation indicates that the decedent wanted to be killed.
- Victim was a bystander: decedent was not the intended target in the incident (e.g., pedestrian walking past a gang fight).
- Victim was a police officer on duty: decedent was a law enforcement officer killed in the line of duty.
- Victim was an intervener assisting a crime victim: decedent was attempting to assist a crime victim at the time of the incident (e.g., a child attempts to intervene and is killed while trying to assist a parent who is being assaulted).
- Victim used a weapon: decedent used a weapon to attack or defend during the course of the incident.
- Intimate partner violence related: incident is related to conflict between current or former intimate partners; includes the death of an intimate partner or nonintimate partner (e.g., child, parent, friend, or law enforcement officer) killed in an incident that originated in a conflict between intimate partners.
- Hate crime: decedent was selected intentionally because of his or her actual or perceived gender, religion, sexual orientation, race, ethnicity, or disability.
- Mentally ill suspect: suspect’s attack on decedent was believed to be the direct result of a mental illness.
- Drive-by shooting: suspect drove near the decedent and fired a weapon while driving.
- Walk-by assault: decedent was killed by a targeted attack (e.g., ambush) where the suspect fled on foot.
- Random violence: decedent was killed in a random act of violence (i.e., an act in which the suspect is not concerned with who is being harmed, just that someone is being harmed).
- Gang related: incident resulted from gang activity or gang rivalry; not used if the decedent was a gang member and the death did not appear to result from gang activity.
- Justifiable self-defense: decedent was killed by a law enforcement officer in the line of duty or by a civilian in legitimate self-defense or in defense of others.

**All Manners of Death (Except Unintentional Firearm)**

- Current depressed mood: decedent was perceived by self or others to be feeling depressed at the time of death.
- Current diagnosed mental health problem: decedent was identified as having a mental health disorder or syndrome listed in the *Diagnostic and Statistical Manual, Version V* (*DSM-V*), with the exception of alcohol and other substance dependence (these are captured in separate variables).
- Type of mental health diagnosis: identifies the type of *DSM-V* diagnosis reported for the decedent.
- Current mental health treatment: decedent was receiving mental health treatment as evidenced by a current prescription for a psychotropic medication, visit or visits to a mental health professional, or participation in a therapy group within the previous 2 months.
- History of ever being treated for mental health problem: decedent was identified as having ever received mental health treatment.
- Alcohol problem: decedent was perceived by self or others to have a problem with, or to be addicted to, alcohol.
- Substance use problem (excludes alcohol): decedent was perceived by self or others to have a problem with, or be addicted to, a substance other than alcohol.
- Other addiction: decedent was perceived by self or others to have an addiction other than to alcohol or other substance (e.g., gambling or sex).
- Family relationship problem: decedent was experiencing problems with a family member, other than an intimate partner.
- Other relationship problem (nonintimate): decedent was experiencing problems with a friend or associate (other than an intimate partner or family member).
- History of child abuse/neglect: as a child, decedent had history of physical, sexual, or psychological abuse; physical (including medical or dental), emotional, or educational neglect; exposure to a violent environment, or inadequate supervision by a caretaker.
- Caretaker abuse/neglect led to death: decedent was experiencing physical, sexual, or psychological abuse; physical (including medical or dental), emotional, or educational neglect; exposure to a violent environment; or inadequate supervision by a caretaker that led to death.
- Perpetrator of interpersonal violence during previous month: decedent perpetrated interpersonal violence during the previous month.
- Victim of interpersonal violence during previous month: decedent was the target of interpersonal violence during the past month.
- Physical fight (two persons, not a brawl): a physical fight between two individuals that resulted in the death of the decedent, who was either involved in the fight, a bystander, or trying to stop the fight.
- Argument or conflict: a specific argument or disagreement led to the victim’s death.
- Precipitated by another crime: incident occurred as the result of another serious crime.
- Nature of crime: the specific type of other crime that occurred during the incident (e.g., robbery or drug trafficking).
- Crime in progress: another serious crime was in progress at the time of the incident.
- Terrorist attack: decedent was injured in a terrorist attack, leading to death.
- Crisis during previous or upcoming 2 weeks: current crisis or acute precipitating event or events that either occurred during the previous 2 weeks or was impending in the following 2 weeks (e.g., a trial for a criminal offense begins the following week). Crises typically are associated with specific circumstance variables (e.g., job problem was a crisis or a financial problem was a crisis).
- Other crisis: a crisis related to a death but not captured by any of the standard circumstances.

**Unintentional Firearm Death**

*Context of Injury*

- Hunting: death occurred any time after leaving home for a hunting trip and before returning home from a hunting trip.
- Target shooting: shooter was aiming for a target and unintentionally hit the decedent; can be at a shooting range or an informal backyard setting (e.g., teenagers shooting at signposts on a fence).
- Loading/unloading gun: gun discharged when the shooter was loading/unloading ammunition.
- Cleaning gun: shooter pulled trigger or gun discharged while cleaning, repairing, assembling, or disassembling gun.
- Showing gun to others: gun was being shown to another person when it discharged or the trigger was pulled.
- Playing with gun: shooter was playing with a gun when it discharged.
- Celebratory firing: shooter fired gun in celebratory manner (e.g., firing into the air at midnight on New Year’s Eve).
- Other context of injury: shooting occurred during some context other than those already described.

*Mechanism of Injury*

- Unintentionally pulled trigger: shooter unintentionally pulled the trigger (e.g., while grabbing the gun or holding it too tightly).
- Thought gun safety was engaged: shooter thought the safety was on and gun would not discharge.
- Thought unloaded/magazine disengaged: shooter thought the gun was unloaded because the magazine was disengaged.
- Thought gun was unloaded: shooter thought the gun was unloaded for other unspecified reason.
- Bullet ricocheted: bullet ricocheted from its intended target and struck the decedent.
- Gun fired due to defect or malfunction: gun had a defect or malfunctioned as determined by a trained firearm examiner.
- Gun fired while holstering: gun was being replaced or removed from holster/clothing.
- Gun was dropped: gun discharged when it was dropped.
- Gun fired while operating safety/lock: shooter unintentionally fired the gun while operating the safety/lock.
- Gun was mistaken for toy: gun was mistaken for a toy and was fired without the user understanding the danger.
- Other mechanism of injury: shooting occurred as the result of a mechanism not already described.

- manner of death (i.e., the intent to cause death [suicide, homicide, legal intervention, unintentional, or undetermined] of the person on whom a fatal injury was inflicted) (Box 1);
- demographic information (e.g., age, sex, and race/ethnicity) of victims and suspects (if applicable);
- method of injury (i.e., the mechanism used to inflict a fatal injury) (Box 2);
- location, date, and time of injury and death;
- toxicology findings (for decedents who were tested);
- circumstances (i.e., the events that preceded and were identified by investigators as relevant and therefore might have contributed to the infliction of a fatal injury) (Box 3);
- whether the decedent was a victim (i.e., a person who died as a result of a violence-related injury) or both a suspect and a victim (i.e., a person believed to have inflicted a fatal injury on a victim who then was fatally injured, such as the perpetrator of a homicide followed by suicide);
- information about any known suspects (i.e., a person or persons believed to have inflicted a fatal injury on a victim);
- incident (i.e., an occurrence in which one or more persons sustained a fatal injury that was linked to a common event or perpetrated by the same suspect or suspects during a 24-hour period); and
- type of incident (i.e., a combination of the manner of death and the number of victims in an incident).

### Circumstances Preceding Death

Circumstances preceding death are defined as the precipitating events that contributed to the infliction of a fatal injury (Box 3). Circumstances are reported on the basis of the content of coroner or medical examiner and law enforcement investigative reports. Certain circumstances are coded to a specific manner of death (e.g., “history of suicide attempt” is collected for suicides or deaths of undetermined intent); other circumstances are coded across all manners of death (e.g., “current diagnosed mental health problem”). The data abstractor selects from a list of potential circumstances and is required to code all circumstances that are known to relate to each incident. If circumstances are not known (e.g., a body was found in the woods with no other details reported), the data abstractor does not endorse circumstances; these deaths are then excluded from the denominator for circumstance values. If either the coroner or medical examiner report or law enforcement report indicates the presence of a circumstance, then the abstractor endorses the circumstance (e.g., if the law enforcement report indicated that a decedent had disclosed an intent to die by suicide, then the circumstance variable “disclosed suicidal intent” is endorsed).

Data abstractors draft two incident narratives: one that summarizes the sequence of events of the incident from the perspective of the coroner or medical examiner report and one that summarizes the sequence of events of the incident from the perspective of the law enforcement report. In addition to briefly summarizing the incident (i.e., the who, what, when, where, and why), the narratives provide supporting information on circumstances that the data abstractor indicated and context for understanding the incident, record information and additional detail that cannot be captured elsewhere, and facilitate data quality control checks on the coding of key variables.

In NVDRS, the circumstance variable “intimate partner violence related” identifies cases in which the homicide or legal intervention death was related to immediate or ongoing conflict or violence between current or former intimate partners. In this report, intimate partner violence–related homicides include victims killed by an intimate partner (e.g., current, former, or unspecified spouse, boyfriend, or girlfriend) and victims killed during an intimate partner violence–related homicide who were not the intimate partner (e.g., children, other family members, friends, or others who might have intervened in intimate partner violence [e.g., first responders or bystanders]).

### Coding Training and Quality Control

Ongoing coding support for data abstractors is provided by CDC through an electronic help desk, monthly conference calls, annual in-person meetings that include coding training for data abstractors, and regular conference calls with individual VDRS programs. In addition, all data abstractors are invited to participate in monthly coding work group calls. VDRS programs can conduct additional abstractor training workshops and activities at their own discretion, including through the use of NVDRS Data Abstractor eLearn Training Modules. An NVDRS coding manual (*7*) with CDC-issued standard guidance on coding criteria and examples for each data element is provided to each VDRS program. Software features to enhance coding reliability include automated validation rules and a hover-over feature containing variable-specific information.

VDRS programs annually are requested to perform reabstractions of a subset of cases using multiple abstractors to identify inconsistencies. Each VDRS program’s data quality is also evaluated by CDC. Before the data release each year, CDC runs a quality control analysis that reviews multiple variables for data inconsistencies, with special focus on abstractor-assigned variables (e.g., method of injury and manner of death). If CDC finds inconsistencies, the VDRS program is notified and asked for a response or correction. VDRS programs must meet CDC standards for completeness of circumstance data to be included in the national data set. VDRS programs must have circumstance information for at least 50% of cases abstracted from either the coroner or medical examiner report or the law enforcement report. However, VDRS programs often far exceed this requirement. For 2017, a total of 87.5% of suicides, homicides, and legal intervention deaths in NVDRS had circumstance data from either the coroner or medical examiner report or the law enforcement report. In addition, core variables that represent demographic characteristics (e.g., age, sex, and race/ethnicity) and manners of death were missing or unknown for <2% of cases. To ensure the final data set has no duplicate records, NVDRS first uses SAS (version 9.4; SAS Institute) to search for any instances of duplicates of a unique identification variable associated with each decedent record. As a second and final check for duplicates, the SAS data set is created with an index that only executes successfully if no duplicates of this identification variable are found.

### Time Frame

VDRS programs are required to begin entering all deaths into the web-based system within 4 months from the date the violent death occurred. VDRS programs then have an additional 16 months from the end of the calendar year in which the violent death occurred to complete each incident record. Although VDRS programs typically meet these requirements, additional details occasionally arrive after a deadline has passed. New incidents also might be identified after the deadline (e.g., a death certificate is revised, new evidence is obtained that changes a manner of death, or an *ICD-10* miscoding is corrected to meet the NVDRS case definition). These additional data are incorporated into NVDRS when analysis files are updated in real time in the web-based system. Eight months after the 16-month data collection period for the 2017 data year, case counts increased by <0.1%.

### Inclusion Criteria

 The inclusion criteria for violent deaths in this report are as follows: 1) cases met the NVDRS case definition for violent death; 2) cases occurred in participating VDRS states, the District of Columbia, or Puerto Rico in 2017; and 3) at least 50% of cases for each included state, district, or territory had circumstance data collected from the coroner or medical examiner report or law enforcement report. All but one eligible VDRS program (Hawaii) met the completeness threshold for circumstances.

Of the participating VDRS programs, 31 states (Alaska, Arizona, Colorado, Connecticut, Delaware, Georgia, Indiana, Iowa, Kansas, Kentucky, Maine, Maryland, Massachusetts, Michigan, Minnesota, Nevada, New Hampshire, New Jersey, New Mexico, New York, North Carolina, Ohio, Oklahoma, Oregon, Rhode Island, South Carolina, Utah, Vermont, Virginia, West Virginia, and Wisconsin) collected information on all violent deaths that occurred in their states in 2017. In addition, data were collected on all violent deaths that occurred in the District of Columbia and Puerto Rico in 2017. Three states (Illinois, Pennsylvania, and Washington) joined NVDRS with plans to collect data on violent deaths for a subset of counties that represented at least 80% of all violent deaths in their state or in counties where at least 1,800 violent deaths occurred. In 2017, these states reported data from a subset of counties that represented at least 80% of violent deaths in their state. These counties represented 79.3% of Illinois’ population, 81.7% of Pennsylvania’s population, and 95.5% of Washington’s population (*8*). California collected data from death certificates for all violent deaths in the state in 2017 (n = 6,715); however, only four counties (Los Angeles, Sacramento, Shasta, and Siskiyou) collected data from all three required sources (i.e., death certificates, coroner or medical examiner reports, and law enforcement reports) and are included in this report (n = 1,866; 27.8%). These four counties represented 30.1% of California’s population (*8*). Because <100% of violent deaths were reported, data from California, Illinois, Pennsylvania, and Washington are not representative of all violent deaths occurring in these four states.

In 2017, the 34 states (including the subset of counties in Illinois, Pennsylvania, and Washington), four California counties, and District of Columbia accounted for 63.5% of the U.S. population (*8*). Puerto Rico collected data on all violent deaths that occurred in the territory in 2017.

### Analyses

This report includes data for violent deaths that occurred in 34 states, four California counties, the District of Columbia, and Puerto Rico in 2017. VDRS program-level data received by CDC as of July 16, 2019, were consolidated and analyzed. The numbers, percentages, and crude rates are presented in aggregate for all deaths by abstractor-assigned manner of death. The suicide rate was calculated using denominators among populations aged >10 years. The rate for other manners of death (e.g., homicide rate and legal intervention death rate) used denominators among populations of all ages. The rates for cells with frequency <20 are not reported because of the instability of those rates. Denominators for the rates for the four states that did not collect statewide data (California, Illinois, Pennsylvania, and Washington) represent only the populations of the counties from which data were collected. The rates could not be calculated for certain variables (e.g., circumstances) because denominators were unknown.

Bridged-race 2017 population estimates were used as denominators in the crude rate calculations for the 34 states, four California counties, and District of Columbia (*9*). For compatible numerators for the rate calculations to be derived, records listing multiple races were recoded to a single race, when possible, using race-bridging methods described by NCHS (https://www.cdc.gov/nchs/nvss/bridged_race.htm) (*10*). The rates specific to race/ethnicity are not available for Puerto Rico because the U.S. Census Bureau estimates for Puerto Rico do not include race or Hispanic origin (*11*). Data for Puerto Rico were analyzed separately. Population estimates by sex and age were used as denominators in the crude rate calculations for Puerto Rico (*12*). 

## Results

### Violent Deaths in 34 States, Four California Counties, and the District of Columbia

For 2017, a total of 34 NVDRS states, four California counties, and the District of Columbia collected data on 45,141 incidents involving 46,389 deaths (Supplementary Table S1, https://stacks.cdc.gov/view/cdc/96308). Suicide (n = 29,454; 63.5%) accounted for the highest rate of violent death (16.3 per 100,000 population aged ≥10 years), followed by homicide (n = 11,550; 24.9%) (5.6 per 100,000 population). Deaths of undetermined intent (n = 4,496; 9.7%), legal intervention deaths (n = 635; 1.4%), and unintentional firearm deaths (n = 254; <1.0%) occurred at lower rates (2.2, 0.3, and 0.1 per 100,000 population, respectively). Deaths by manner that include statewide counts and the rates for California are available (Supplementary Table S2, https://stacks.cdc.gov/view/cdc/96308).

### Suicides

#### Sex, Age Group, and Race/Ethnicity

For 2017, a total of 34 NVDRS states, four California counties, and the District of Columbia collected data on 29,405 incidents involving 29,454 suicide deaths among persons aged ≥10 years. Overall, the suicide rate was 16.3 per 100,000 population aged ≥10 years (Table 1).

**TABLE 1:** Number, percentage,* and rate^†^ of suicides among persons aged ≥10 years,^§^ by decedent’s sex,^¶^ age group, race/ethnicity, method used, and location where injury occurred — National Violent Death Reporting System, 34 states,** four California counties, and the District of Columbia, 2017

**Characteristic**	Male		Female		Total	
	**No. (%)**	**Rate**	**No. (%)**	**Rate**	**No. (%)**	**Rate**
**Age group (yrs)**						
10–14	215 (<1.0)	3.2	112 (1.7)	1.8	**327 (1.1)**	**2.5**
15–19	1,225 (5.4)	18.0	348 (5.3)	5.3	**1,573 (5.3)**	**11.8**
20–24	1,949 (8.5)	27.3	429 (6.5)	6.3	**2,378 (8.1)**	**17.0**
25–29	2,082 (9.1)	27.8	494 (7.5)	6.8	**2,576 (8.7)**	**17.5**
30–34	1,879 (8.2)	26.9	541 (8.2)	7.8	**2,421 (8.2)**	**17.4**
35–44	3,460 (15.1)	27.0	1,144 (17.3)	8.8	**4,604 (15.6)**	**17.9**
45–54	4,005 (17.5)	30.0	1,412 (21.4)	10.3	**5,417 (18.4)**	**20.0**
55–64	3,751 (16.4)	28.8	1,201 (18.2)	8.6	**4,952 (16.8)**	**18.3**
65–74	2,219 (9.7)	25.2	617 (9.4)	6.1	**2,836 (9.6)**	**15.0**
75–84	1,371 (6.0)	34.3	217 (3.3)	4.1	**1,588 (5.4)**	**17.2**
≥85	695 (3.0)	48.5	82 (1.2)	3.0	**777 (2.6)**	**18.7**
Unknown	5 (<1.0)	—^††^	0 (0.0)	—	**5 (<1.0)**	**—**
**Race/Ethnicity**						
White, non-Hispanic	18,697 (81.8)	31.5	5,366 (81.3)	8.7	**24,063 (81.7)**	**19.9**
Black, non-Hispanic	1,534 (6.7)	13.9	415 (6.3)	3.3	**1,949 (6.6)**	**8.3**
American Indian/Alaska Native, non-Hispanic	352 (1.5)	43.0	125 (1.9)	14.4	**477 (1.6)**	**28.3**
Asian/Pacific Islander	608 (2.7)	12.2	284 (4.3)	5.1	**892 (3.0)**	**8.5**
Hispanic^§§^	1,602 (7.0)	13.0	394 (6.0)	3.3	**1,997 (6.8)**	**8.2**
Other	60 (<1.0)	—	12 (<1.0)	—	**72 (<1.0)**	**—**
Unknown	3 (<1.0)	—	1 (<1.0)	—	**4 (<1.0)**	**—**
**Method**						
Firearm	12,292 (53.8)	13.9	1,898 (28.8)	2.1	**14,190 (48.2)**	**7.8**
Hanging/strangulation/suffocation	6,759 (29.6)	7.6	1,999 (30.3)	2.2	**8,758 (29.7)**	**4.8**
Poisoning	1,923 (8.4)	2.2	2,046 (31.0)	2.2	**3,970 (13.5)**	**2.2**
Fall	580 (2.5)	0.7	197 (3.0)	0.2	**777 (2.6)**	**0.4**
Sharp instrument	443 (1.9)	0.5	111 (1.7)	0.1	**554 (1.9)**	**0.3**
Motor vehicle (e.g., bus, motorcycle, or other transport vehicle)	372 (1.6)	0.4	122 (1.8)	0.1	**494 (1.7)**	**0.3**
Drowning	186 (<1.0)	0.2	103 (1.6)	0.1	**289 (<1.0)**	**0.2**
Fire/burns	71 (<1.0)	<0.1	57 (<1.0)	<0.1	**128 (<1.0)**	**<0.1**
Blunt instrument	13 (<1.0)	—	4 (<1.0)	—	**17 (<1.0)**	**—**
Intentional neglect	5 (<1.0)	—	4 (<1.0)	—	**9 (<1.0)**	**—**
Other (single method)	50 (<1.0)	—	10 (<1.0)	—	**60 (<1.0)**	**—**
Unknown	162 (<1.0)	—	46 (<1.0)	—	**208 (<1.0)**	**—**
**Location**						
House/apartment	16,141 (70.6)	18.2	5,207 (78.9)	5.6	**21,349 (72.5)**	**11.8**
Motor vehicle	1,199 (5.2)	1.4	252 (3.8)	0.3	**1,451 (4.9)**	**0.8**
Natural area	1,199 (5.2)	1.4	205 (3.1)	0.2	**1,404 (4.8)**	**0.8**
Street/highway	618 (2.7)	0.7	103 (1.6)	0.1	**721 (2.4)**	**0.4**
Hotel/motel	468 (2.0)	0.5	177 (2.7)	0.2	**645 (2.2)**	**0.4**
Park/playground/sports or athletic area	432 (1.9)	0.5	82 (1.2)	<0.1	**514 (1.7)**	**0.3**
Parking lot/public garage/public transport	406 (1.8)	0.5	72 (1.1)	<0.1	**478 (1.6)**	**0.3**
Other location^¶¶^	1,824 (8.0)	—	336 (5.1)	—	**2,160 (7.3)**	**—**
Unknown	569 (2.5)	—	163 (2.5)	—	**732 (2.5)**	**—**
**Total**	**22,856 (100)**	**25.8**	**6,597 (100)**	**7.1**	**29,454 (100)**	**16.3**

* Percentages might not total 100% due to rounding.

^†^ Per 100,000 population.

^§^ Suicide is not reported for decedents aged <10 years, as per standard in the suicide prevention literature. Denominators for the suicide rates represent the total population aged ≥10 years.

^¶^ Sex was unknown for one decedent.

** Alaska, Arizona, Colorado, Connecticut, Delaware, Georgia, Illinois, Indiana, Iowa, Kansas, Kentucky, Maine, Maryland, Massachusetts, Michigan, Minnesota, Nevada, New Hampshire, New Jersey, New Mexico, New York, North Carolina, Ohio, Oklahoma, Oregon, Pennsylvania, Rhode Island, South Carolina, Utah, Vermont, Virginia, Washington, West Virginia, and Wisconsin. Illinois, Pennsylvania, and Washington collected data on ≥80% of violent deaths in their state, in accordance with requirements under which these states were funded. Data for California are for violent deaths that occurred in four counties (Los Angeles, Sacramento, Shasta, and Siskiyou). Denominators for the rates for these four states (California, Illinois, Pennsylvania, and Washington) represent only the populations of the counties from which the data were collected.

^††^ Rate is not reported when number of decedents is <20 or when characteristic response is “other” or “unknown.”

^§§^ Includes persons of any race.

^¶¶^ Other location includes (in descending order) jail/prison, bridge, railroad tracks, commercial/retail area, farm, hospital or medical facility, supervised residential facility, cemetery/graveyard/other burial ground, industrial or construction area, preschool/school/college/school bus, office building, abandoned house/building/warehouse, synagogue/church/temple, bar/nightclub, and other unspecified location.

The suicide rate for males was 3.6 times the rate for females (25.8 versus 7.1 per 100,000 population, respectively) (Table 1). The suicide rate for males ranged from 1.8 to 16.2 times the rate for females across age groups and from 2.4 to 4.2 times the rate for females across racial/ethnic groups. Adults aged 45–54 years, 55–64 years, and ≥85 years had the highest rates of suicide across age groups (20.0, 18.3, and 18.7 per 100,000 population, respectively). Non-Hispanic Whites accounted for the majority (81.7%) of suicides; however, non-Hispanic American Indians/Alaska Natives had the highest rate of suicide (28.3 per 100,000 population) among racial/ethnic groups.

Among males, nearly half (49.0%) of suicide decedents were men aged 35–64 years (Table 1). Men aged ≥85 years had the highest rate of suicide (48.5 per 100,000 population), followed by men aged 75–84 and 45–54 years (34.3 and 30.0 per 100,000 population, respectively). Non-Hispanic American Indian/Alaska Native males had the highest rate of suicide (43.0 per 100,000 population), followed by non-Hispanic White males (31.5 per 100,000 population). The rate of suicide for non-Hispanic American Indian/Alaska Native males was 3.5 times the rate for males with the lowest rate, Asian/Pacific Islanders (12.2 per 100,000 population).

Among females, women aged 35–64 years accounted for more than half (56.9%) of suicides (Table 1). Women aged 45–54 years had the highest rate of suicide (10.3 per 100,000 population). The suicide rate was highest among non-Hispanic American Indian/Alaska Native females (14.4 per 100,000 population), followed by non-Hispanic White (8.7 per 100,000 population), Asian/Pacific Islander (5.1 per 100,000 population), non-Hispanic Black (3.3 per 100,000 population), and Hispanic (3.3 per 100,000 population) females. The rate of suicide for non-Hispanic American Indian/Alaska Native females was 4.4 times the rate for females with the lowest rates (non-Hispanic Black and Hispanic females).

#### Method and Location of Injury

A firearm was used in approximately half (48.2%) of suicides, followed by hanging/strangulation/suffocation (29.7%) and poisoning (13.5%) (rates of 7.8, 4.8, and 2.2 per 100,000 population, respectively) (Table 1). Among males, the most common method of injury was a firearm (53.8%), followed by hanging/strangulation/suffocation (29.6%). Among females, poisoning (31.0%) was the most common method of injury and was used in approximately equal proportions as hanging/strangulation/suffocation (30.3%) and a firearm (28.8%). The most common location of suicide was a house/apartment (72.5%), followed by a motor vehicle (4.9%), a natural area (4.8%), a street/highway (2.4%), and a hotel/motel (2.2%).

#### Toxicology Results of Decedent

Tests for alcohol were conducted for 52.7% of suicide decedents (Table 2). Among those with positive results for alcohol (40.2%), 65.3% had a blood alcohol concentration (BAC) ≥0.08 g/dL. Tests for amphetamines, antidepressants, benzodiazepines, cocaine, marijuana, and opioids were conducted for 39.9%, 26.7%, 40.3%, 41.1%, 34.0%, and 43.5% of decedents, respectively. Results for opioids (including illicit and prescription) were positive in 25.2% of decedents tested for these substances. Results for amphetamines, cocaine, and marijuana were positive in 12.2%, 7.7%, and 22.6% of decedents tested, respectively. Of those tested for antidepressants, 40.2% had positive results at the time of death, and 28.6% of those tested for benzodiazepines had positive results. Carbon monoxide was tested for a substantially smaller proportion of decedents (6.7%) but was identified in nearly one third of those decedents (28.8%).

**TABLE 2:** Number* and percentage of suicide decedents tested for alcohol and drugs and whose results were positive,^†^ by toxicology variables — National Violent Death Reporting System, 34 states,^§^ four California counties, and the District of Columbia, 2017

Toxicology variable	Tested	Positive
	No. (%)	No. (%)
Blood alcohol concentration^¶^	15,518 (52.7)	6,237 (40.2)
Alcohol <0.08 g/dL		1,780 (28.5)
Alcohol ≥0.08 g/dL		4,070 (65.3)
Alcohol positive, level unknown		387 (6.2)
Amphetamines	11,766 (39.9)	1,435 (12.2)
Anticonvulsants	6,186 (21.0)	1,045 (16.9)
Antidepressants	7,864 (26.7)	3,158 (40.2)
Antipsychotics	6,309 (21.4)	770 (12.2)
Barbiturates	9,735 (33.1)	255 (2.6)
Benzodiazepines	11,858 (40.3)	3,392 (28.6)
Carbon monoxide	1,976 (6.7)	570 (28.8)
Cocaine	12,111 (41.1)	934 (7.7)
Marijuana	10,014 (34.0)	2,266 (22.6)
Muscle relaxants	6,328 (21.5)	446 (7.0)
Opioids	12,803 (43.5)	3,220 (25.2)
Other drugs/substances**	6,818 (23.1)	5,670 (83.2)

* Number of suicide decedents = 29,454.

^†^ Percentage of decedents tested for toxicology.

^§^ Alaska, Arizona, Colorado, Connecticut, Delaware, Georgia, Illinois, Indiana, Iowa, Kansas, Kentucky, Maine, Maryland, Massachusetts, Michigan, Minnesota, Nevada, New Hampshire, New Jersey, New Mexico, New York, North Carolina, Ohio, Oklahoma, Oregon, Pennsylvania, Rhode Island, South Carolina, Utah, Vermont, Virginia, Washington, West Virginia, and Wisconsin. Illinois, Pennsylvania, and Washington collected data on ≥80% of violent deaths in their state, in accordance with requirements under which these states were funded. Data for California are for violent deaths that occurred in four counties (Los Angeles, Sacramento, Shasta, and Siskiyou).

^¶^ Blood alcohol concentration of ≥0.08 g/dL is over the legal limit in all states and is used as the standard for intoxication.

** Other drugs/substances indicated whether any results for other drugs/substances were positive; levels for these drugs/substances are not measured.

#### Precipitating Circumstances

Circumstances were identified in 26,839 (91.1%) of suicides (Table 3). Overall, a mental health problem was the most common circumstance, with approximately half (49.8%) of decedents having had a current diagnosed mental health problem and 34.9% experiencing a depressed mood at the time of death. Among the 13,378 decedents with a current diagnosed mental health problem, depression/dysthymia (73.6%), anxiety disorder (18.9%), and bipolar disorder (15.1%) were the most common diagnoses. Among suicide decedents, 27.5% were receiving mental health treatment at the time of death.

**TABLE 3:** Number* and percentage^†^ of suicides among persons aged ≥10 years,^§^ by decedent’s sex and precipitating circumstances — National Violent Death Reporting System, 34 states,^¶^ four California counties, and the District of Columbia, 2017

Precipitating circumstance	Male	Female	Total
	No. (%)	No. (%)	No. (%)
**Mental health/Substance use**			
Current diagnosed mental health problem**	9,352 (45.2)	4,026 (65.7)	**13,378 (49.8)**
Depression/dysthymia	6,758 (72.3)	3,091 (76.8)	**9,850 (73.6)**
Anxiety disorder	1,604 (17.2)	922 (22.9)	**2,526 (18.9)**
Bipolar disorder	1,264 (13.5)	759 (18.9)	**2,023 (15.1)**
Schizophrenia	562 (6.0)	166 (4.1)	**728 (5.4)**
PTSD	550 (5.9)	169 (4.2)	**719 (5.4)**
ADD/ADHD	301 (3.2)	54 (1.3)	**355 (2.7)**
OCD	44 (<1.0)	19 (<1.0)	**63 (<1.0)**
Eating disorder	5 (<1.0)	31 (<1.0)	**36 (<1.0)**
Other	578 (6.2)	202 (5.0)	**780 (5.8)**
Unknown	888 (9.5)	367 (9.1)	**1,255 (9.4)**
History of ever being treated for a mental health problem	6,833 (33.0)	3,155 (51.5)	**9,988 (37.2)**
Current depressed mood	7,192 (34.7)	2,167 (35.3)	**9,359 (34.9)**
Current mental health treatment	4,889 (23.6)	2,487 (40.6)	**7,376 (27.5)**
Alcohol problem	3,974 (19.2)	959 (15.6)	**4,933 (18.4)**
Substance use problem (excludes alcohol)	3,594 (17.4)	1,176 (19.2)	**4,770 (17.8)**
Other addiction (e.g., gambling or sex)	150 (<1.0)	41 (<1.0)	**191 (<1.0)**
**Interpersonal**			
Intimate partner problem	5,759 (27.8)	1,497 (24.4)	**7,256 (27.0)**
Family relationship problem	1,908 (9.2)	667 (10.9)	**2,575 (9.6)**
Other death of family member or friend	1,360 (6.6)	463 (7.6)	**1,823 (6.8)**
Suicide of family member or friend	487 (2.4)	186 (3.0)	**673 (2.5)**
Perpetrator of interpersonal violence during past month	603 (2.9)	48 (<1.0)	**651 (2.4)**
Other relationship problem (nonintimate)	468 (2.3)	165 (2.7)	**633 (2.4)**
Victim of interpersonal violence during past month	64 (<1.0)	84 (1.4)	**148 (<1.0)**
**Life stressor**			
Crisis during previous or upcoming 2 weeks	6,740 (32.6)	1,668 (27.2)	**8,408 (31.3)**
Physical health problem	4,527 (21.9)	1,279 (20.9)	**5,806 (21.6)**
Argument or conflict	3,356 (16.2)	1,000 (16.3)	**4,356 (16.2)**
Job problem	2,178 (10.5)	394 (6.4)	**2,572 (9.6)**
Financial problem	1,864 (9.0)	440 (7.2)	**2,304 (8.6)**
Recent criminal legal problem	1,941 (9.4)	231 (3.8)	**2,172 (8.1)**
Eviction or loss of home	815 (3.9)	217 (3.5)	**1,032 (3.8)**
Noncriminal legal problem	642 (3.1)	197 (3.2)	**839 (3.1)**
School problem	326 (1.6)	98 (1.6)	**424 (1.6)**
History of child abuse/neglect	185 (<1.0)	126 (2.1)	**311 (1.2)**
Physical fight (two persons, not a brawl)	220 (1.1)	30 (<1.0)	**250 (<1.0)**
Traumatic anniversary	155 (<1.0)	63 (1.0)	**218 (<1.0)**
Caretaker abuse/neglect led to suicide	18 (<1.0)	20 (<1.0)	**38 (<1.0)**
Exposure to disaster	25 (<1.0)	2 (<1.0)	**27 (<1.0)**
**Crime and criminal activity**			
Precipitated by another crime	937 (4.5)	82 (1.3)	**1,019 (3.8)**
Crime in progress^††^	311 (33.2)	15 (18.3)	**326 (32.0)**
Terrorist attack	0 (0.0)	0 (0.0)	**0 (0.0)**
**Suicide event**			
Left a suicide note	6,548 (31.6)	2,478 (40.4)	**9,026 (33.6)**
History of suicidal thoughts or plans	6,621 (32.0)	2,307 (37.6)	**8,928 (33.3)**
History of suicide attempt or attempts	3,350 (16.2)	2,018 (32.9)	**5,368 (20.0)**
**Suicide disclosure**			
Disclosed suicidal intent	5,110 (24.7)	1,481 (24.2)	**6,591 (24.6)**
Disclosed intent to whom^§§^			
Previous or current intimate partner	1,972 (38.6)	489 (33.0)	**2,462 (37.3)**
Other family member	1,479 (28.9)	480 (32.4)	**1,959 (29.7)**
Friend/colleague	661 (12.9)	196 (13.2)	**857 (13.0)**
Health care worker	198 (3.9)	90 (6.1)	**288 (4.4)**
Neighbor	62 (1.2)	26 (1.8)	**88 (1.3)**
Other person	425 (8.3)	107 (7.2)	**532 (8.1)**
Unknown	313 (6.1)	93 (6.3)	**406 (6.2)**
**Total^¶¶^**	**20,706 (90.6)**	**6,132 (93.0)**	**26,839 (91.1)**

**Abbreviations:** ADD/ADHD = attention deficit disorder/attention deficit hyperactivity disorder; OCD = obsessive-compulsive disorder; PTSD = posttraumatic stress disorder.

* Includes suicides with one or more precipitating circumstances. More than one circumstance could have been present per decedent.

^†^ Denominator includes those suicides with one or more precipitating circumstances. The sums of percentages in columns exceed 100% because more than one circumstance could have been present per decedent.

^§^ Suicide is not reported for decedents aged <10 years, as per standard in the suicide prevention literature. Denominators for the suicide rates represent the total population aged ≥10 years.

^¶^ Alaska, Arizona, Colorado, Connecticut, Delaware, Georgia, Illinois, Indiana, Iowa, Kansas, Kentucky, Maine, Maryland, Massachusetts, Michigan, Minnesota, Nevada, New Hampshire, New Jersey, New Mexico, New York, North Carolina, Ohio, Oklahoma, Oregon, Pennsylvania, Rhode Island, South Carolina, Utah, Vermont, Virginia, Washington, West Virginia, and Wisconsin. Illinois, Pennsylvania, and Washington collected data on ≥80% of violent deaths in their state, in accordance with requirements under which these states were funded. Data for California are for violent deaths that occurred in four counties (Los Angeles, Sacramento, Shasta, and Siskiyou).

** Includes decedents with one or more diagnosed current mental health problems; therefore, sums of percentages for the diagnosed conditions exceed 100%. Denominator includes the number of decedents with one or more current diagnosed mental health problems.

^††^ Denominator includes those decedents involved in an incident that was precipitated by another crime.

^§§^ Denominator includes decedents who disclosed intent.

^¶¶^ Circumstances were unknown for 2,615 decedents (2,150 males and 465 females); total number of suicide decedents = 29,454 (22,856 males, 6,597 females, and one unknown).

Alcohol and other substance use problems were reported for 18.4% and 17.8% of suicide decedents, respectively (Table 3). A recent or impending crisis during the previous or upcoming 2 weeks (31.3%), intimate partner problem (27.0%), physical health problem (21.6%), and argument or conflict (16.2%) were the most commonly reported precipitating circumstances. Among other circumstances related to the suicide, 33.6% of decedents left a suicide note, 33.3% had a history of suicidal thoughts or plans, 20.0% had a history of previous suicide attempts, and 24.6% had disclosed suicidal intent to another person. Of those who disclosed intent, the greatest proportion of disclosures was to a previous or current intimate partner (37.3%), followed by a family member other than an intimate partner (29.7%).

When examining known circumstances by sex, a larger percentage of female decedents had a current diagnosed mental health problem than did male decedents (65.7% versus 45.2%, respectively) (Table 3). Similar percentages of male and female suicide decedents had a depressed mood at the time of death (34.7% and 35.3%, respectively). A larger percentage of female than male decedents was known to have been receiving mental health treatment at the time of death (40.6% versus 23.6%, respectively). Suicide events, including leaving a suicide note, history of suicidal thoughts or plans, and history of suicide attempts, occurred more frequently and at higher rates among females than males.

### Homicides

#### Sex, Age Group, and Race/Ethnicity

For 2017, a total of 34 NVDRS states, four California counties, and the District of Columbia collected data on 10,798 incidents involving 11,550 homicide deaths. Overall, the homicide rate was 5.6 per 100,000 population (Table 4).

**TABLE 4:** Number, percentage,* and rate^†^ of homicides, by decedent’s sex, age group, race/ethnicity, method used, location where injury occurred, and victim-suspect relationship — National Violent Death Reporting System, 34 states,^§^ four California counties, and the District of Columbia, 2017

Characteristic	Male		Female		Total	
	No. (%)	Rate	No. (%)	Rate	No. (%)	Rate
**Age group (yrs)**						
<1	107 (1.2)	8.6	73 (3.1)	6.2	**180 (1.6)**	**7.4**
1–4	118 (1.3)	2.3	77 (3.3)	1.6	**195 (1.7)**	**2.0**
5–9	46 (<1.0)	0.7	41 (1.7)	0.7	**87 (<1.0)**	**0.7**
10–14	63 (<1.0)	1.0	39 (1.7)	0.6	**102 (<1.0)**	**0.8**
15–19	975 (10.6)	14.3	147 (6.3)	2.3	**1,122 (9.7)**	**8.4**
20–24	1,608 (17.5)	22.5	250 (10.7)	3.7	**1,858 (16.1)**	**13.3**
25–29	1,612 (17.5)	21.5	272 (11.6)	3.8	**1,884 (16.3)**	**12.8**
30–34	1,159 (12.6)	16.6	255 (10.9)	3.7	**1,414 (12.2)**	**10.2**
35–44	1,521 (16.5)	11.9	386 (16.4)	3.0	**1,907 (16.5)**	**7.4**
45–54	1,016 (11.0)	7.6	328 (14.0)	2.4	**1,344 (11.6)**	**5.0**
55–64	628 (6.8)	4.8	231 (9.8)	1.7	**859 (7.4)**	**3.2**
65–74	229 (2.5)	2.6	134 (5.7)	1.3	**363 (3.1)**	**1.9**
75–84	84 (<1.0)	2.1	76 (3.2)	1.5	**160 (1.4)**	**1.7**
≥85	36 (<1.0)	2.5	37 (1.6)	1.4	**73 (<1.0)**	**1.8**
Unknown	1 (<1.0)	—^¶^	1 (<1.0)	—	**2 (<1.0)**	**—**
**Race/Ethnicity**						
White, non-Hispanic	2,106 (22.9)	3.2	1,119 (47.7)	1.6	**3,225 (27.9)**	**2.4**
Black, non-Hispanic	5,403 (58.7)	41.3	785 (33.4)	5.5	**6,188 (53.6)**	**22.6**
American Indian/Alaska Native, non-Hispanic	146 (1.6)	15.1	48 (2.0)	4.7	**194 (1.7)**	**9.8**
Asian/Pacific Islander	129 (1.4)	2.3	66 (2.8)	1.1	**195 (1.7)**	**1.6**
Hispanic**	1,390 (15.1)	9.3	318 (13.5)	2.2	**1,708 (14.8)**	**5.8**
Other	27 (<1.0)	—	9 (<1.0)	—	**36 (<1.0)**	**—**
Unknown	2 (<1.0)	—	2 (<1.0)	—	**4 (<1.0)**	**—**
**Method**						
Firearm	7,125 (77.4)	7.0	1,293 (55.1)	1.2	**8,418 (72.9)**	**4.1**
Sharp instrument	885 (9.6)	0.9	351 (15.0)	0.3	**1,236 (10.7)**	**0.6**
Blunt instrument	321 (3.5)	0.3	182 (7.8)	0.2	**503 (4.4)**	**0.2**
Personal weapons (e.g., hands, feet, or fists)	358 (3.9)	0.4	130 (5.5)	0.1	**488 (4.2)**	**0.2**
Hanging/strangulation/suffocation	112 (1.2)	0.1	176 (7.5)	0.2	**288 (2.5)**	**0.1**
Motor vehicle (e.g., bus, motorcycle, other transport vehicle)	83 (<1.0)	<0.1	34 (1.4)	<0.1	**117 (1.0)**	**<0.1**
Fire/burns	54 (<1.0)	<0.1	33 (1.4)	<0.1	**87 (<1.0)**	**<0.1**
Poisoning	17 (<1.0)	—	18 (<1.0)	—	**35 (<1.0)**	**<0.1**
Intentional neglect	16 (<1.0)	—	18 (<1.0)	—	**34 (<1.0)**	**<0.1**
Fall	16 (<1.0)	—	11 (<1.0)	—	**27 (<1.0)**	**<0.1**
Drowning	14 (<1.0)	—	3 (<1.0)	—	**17 (<1.0)**	**—**
Other (single method)	33 (<1.0)	—	25 (1.1)	—	**58 (<1.0)**	**—**
Unknown	169 (1.8)	—	73 (3.1)	—	**242 (2.1)**	**—**
**Location**						
House/apartment	3,647 (39.6)	3.6	1,543 (65.7)	1.5	**5,190 (44.9)**	**2.5**
Street/highway	2,535 (27.5)	2.5	202 (8.6)	0.2	**2,737 (23.7)**	**1.3**
Motor vehicle	933 (10.1)	0.9	167 (7.1)	0.2	**1,100 (9.5)**	**0.5**
Parking lot/public garage/public transport	423 (4.6)	0.4	48 (2.0)	<0.1	**471 (4.1)**	**0.2**
Commercial/retail area	352 (3.8)	0.4	42 (1.8)	<0.1	**394 (3.4)**	**0.2**
Natural area	140 (1.5)	0.1	49 (2.1)	<0.1	**189 (1.6)**	**<0.1**
Bar/nightclub	159 (1.7)	0.2	7 (<1.0)	—	**166 (1.4)**	**<0.1**
Park/playground/sports or athletic area	120 (1.3)	0.1	14 (<1.0)	—	**134 (1.2)**	**<0.1**
Other location^††^	446 (4.8)	—	148 (6.3)	—	**594 (5.1)**	**—**
Unknown	448 (4.9)	—	127 (5.4)	—	**575 (5.0)**	**—**
**Relationship of victim to suspect^§§^**						
Acquaintance/friend	1,066 (32.2)	1.1	170 (10.7)	0.2	**1,236 (25.2)**	**0.6**
Spouse/intimate partner (current or former)	242 (7.3)	0.2	781 (49.2)	0.7	**1,023 (20.9)**	**0.5**
Other person, known to victim	697 (21.0)	—	109 (6.9)	—	**806 (16.4)**	**—**
Stranger	541 (16.3)	0.5	131 (8.2)	0.1	**672 (13.7)**	**0.3**
Child^¶¶^	211 (6.4)	0.2	133 (8.4)	0.1	**344 (7.0)**	**0.2**
Other relative	209 (6.3)	0.2	92 (5.8)	<0.1	**301 (6.1)**	**0.2**
Parent^¶¶^	147 (4.4)	0.2	133 (8.4)	0.1	**280 (5.7)**	**0.1**
Rival gang member	107 (3.2)	0.1	7 (<1.0)	—	**114 (2.3)**	**<0.1**
Other relationship***	93 (2.7)	—	33 (2.2)	—	**126 (2.5)**	**—**
**Total**	**9,203 (100)**	**9.1**	**2,347 (100)**	**2.2**	**11,550 (100)**	**5.6**

* Percentages might not total 100% due to rounding.

^†^ Per 100,000 population.

^§^ Alaska, Arizona, Colorado, Connecticut, Delaware, Georgia, Illinois, Indiana, Iowa, Kansas, Kentucky, Maine, Maryland, Massachusetts, Michigan, Minnesota, Nevada, New Hampshire, New Jersey, New Mexico, New York, North Carolina, Ohio, Oklahoma, Oregon, Pennsylvania, Rhode Island, South Carolina, Utah, Vermont, Virginia, Washington, West Virginia, and Wisconsin. Illinois, Pennsylvania, and Washington collected data on ≥80% of violent deaths in their state, in accordance with requirements under which these states were funded. Data for California are for violent deaths that occurred in four counties (Los Angeles, Sacramento, Shasta, and Siskiyou). Denominators for the rates for these four states (California, Illinois, Pennsylvania, and Washington) represent only the populations of the counties from which the data were collected.

^¶^ Rates are not reported when number of decedents is <20 or when characteristic response is “other” or “unknown.”

** Includes persons of any race.

^††^ Other location includes (in descending order) hotel/motel, jail/prison, abandoned house/building/warehouse, hospital or medical facility, supervised residential facility, office building, preschool/school/college/school bus, industrial or construction area, farm, railroad tracks, synagogue/church/temple, bridge, cemetery/graveyard/other burial ground, and other unspecified location.

^§§^ Percentage is based on the number of homicide decedents with a known victim-suspect relationship (n = 4,902 [42.4%]; 3,313 [36.0%] males and 1,589 [67.7%] females); victim-suspect relationship was unknown for 6,648 decedents.

^¶¶^ Includes adoptive family members (e.g., adopted child), stepfamily members (e.g., stepparent), and foster family members (e.g., foster child).

*** Other relationship includes (in descending order) child of suspect’s boyfriend/girlfriend (e.g., child killed by their mother’s boyfriend), intimate partner of suspect’s parent (e.g., teenager kills their mother’s boyfriend), and victim was law enforcement officer fatally injured in the line of duty.

The homicide rate was highest among adults aged 20–24 years and was higher among males than among females across all age groups, except for those aged 5–9 years (rates were 0.7 per 100,000 population for both boys and girls aged 5–9 years) (Table 4). The homicide rate for men aged 20–24 years was six times the rate for women aged 20–24 years. Among males, the rate of homicide was highest among those aged 20–24 years and 25–29 years (22.5 and 21.5 per 100,000 population, respectively). Among females, the rate of homicide was highest among infants aged <1 year (6.2 per 100,000 population). The overall homicide rate for infants aged <1 year (7.4 per 100,000 population) was 3.7 times the overall rate for children aged 1–4 years (2.0 per 100,000 population) and 10.6 times the rate for children aged 5–9 years (0.7 per 100,000 population).

Non-Hispanic Black males accounted for 58.7% of male homicide victims and approximately half (46.8%) of all homicides (Table 4). Non-Hispanic Black males had the highest rate of homicide across any racial/ethnic group (41.3 per 100,000 population); this rate was 18.0 times the rate for Asian/Pacific Islander males (2.3 per 100,000 population), 12.9 times the rate for non-Hispanic White males (3.2 per 100,000 population), 4.4 times the rate for Hispanic males (9.3 per 100,000 population), and 2.7 times the rate for non-Hispanic American Indian/Alaska Native males (15.1 per 100,000 population).

Among females, the homicide rate was highest among non-Hispanic Blacks (5.5 per 100,000 population) (Table 4), followed by non-Hispanic American Indians/Alaska Natives (4.7 per 100,000 population), Hispanics (2.2 per 100,000 population), non-Hispanic Whites (1.6 per 100,000 population), and Asians/Pacific Islanders (1.1 per 100,000 population).

#### Method, Location of Injury, and Victim-Suspect Relationship

A firearm was used in 72.9% of homicides, followed by sharp instrument (10.7%), blunt instrument (4.4%), personal weapon (e.g., hands, feet, or fists) (4.2%), and hanging/strangulation/suffocation (2.5%) (Table 4). No other known method was used in >1% of homicides. A firearm was the most common method of injury for both males and females (77.4% and 55.1%, respectively); however, the firearm homicide rate for males was 5.8 times the rate for females (7.0 versus 1.2 per 100,000 population, respectively). A larger proportion of homicides among females than males involved a sharp instrument (15.0% versus 9.6%, respectively), blunt instrument (7.8% versus 3.5%, respectively), hanging/strangulation/suffocation (7.5% versus 1.2%, respectively), and personal weapon (5.5% versus 3.9%, respectively). A house/apartment was the most common location of homicide (44.9%), followed by a street/highway (23.7%), a motor vehicle (9.5%), and a parking lot/public garage/public transport (4.1%). However, a larger proportion of homicides among females than among males occurred at a house/apartment (65.7% versus 39.6%, respectively), whereas a larger proportion of homicides among males than among females occurred on a street/highway (27.5% versus 8.6%, respectively).

The relationship of the victim to the suspect was known for 42.4% of homicides (36.0% of males and 67.7% of females) (Table 4). For males, when the relationship was known, the victim-suspect relationship was most often an acquaintance/friend (32.2%); other person known to the victim but the exact nature of the relationship was unclear (21.0%); a stranger (16.3%); or a current or former intimate partner (7.3%). For females, when the relationship was known, approximately half (49.2%) were a current or former intimate partner, followed by acquaintance/friend (10.7%), child (8.4%), parent (8.4%), and stranger (8.2%).

#### Precipitating Circumstances

Precipitating circumstances were identified in 80.5% of homicides (Table 5). Approximately one in three homicides with known circumstances was precipitated by an argument or conflict (31.3%). Homicides also were commonly precipitated by another crime (28.2%); in 62.8% of those cases, the crime was in progress at the time of the incident. The type of crime most frequently precipitating these homicides was assault/homicide (40.2%), robbery (34.9%), drug trade** (15.6%), burglary (12.9%), motor vehicle theft (3.7%), rape/sexual assault (2.6%), and arson (2.0%) (Supplementary Table S10, https://stacks.cdc.gov/view/cdc/96308). In 14.9% of homicides with known circumstances, intimate partner violence was identified as a contributing factor (Table 5). A physical fight between two persons (13.4%) and drug involvement (11.7%) were other common precipitating circumstances.

**TABLE 5:** Number* and percentage^†^ of homicides, by decedent’s sex and precipitating circumstances — National Violent Death Reporting System, 34 states,^§^ four California counties, and the District of Columbia, 2017

Precipitating circumstance	Male	Female	Total
	No. (%)	No. (%)	No. (%)
**Mental health/Substance use**			
Substance use problem (excludes alcohol)	1,095 (15.1)	262 (12.8)	**1,357 (14.6)**
Current diagnosed mental health problem	336 (4.6)	187 (9.1)	**523 (5.6)**
History of ever being treated for a mental health problem	251 (3.5)	131 (6.4)	**382 (4.1)**
Alcohol problem	321 (4.4)	59 (2.9)	**380 (4.1)**
Current mental health treatment	146 (2.0)	100 (4.9)	**246 (2.6)**
Current depressed mood	30 (<1.0)	23 (1.1)	**53 (<1.0)**
Other addiction (e.g., gambling or sex)	14 (<1.0)	1 (<1.0)	**15 (<1.0)**
**Interpersonal**			
Intimate partner violence related	526 (7.3)	856 (41.9)	**1,382 (14.9)**
Family relationship problem	350 (4.8)	198 (9.7)	**548 (5.9)**
Other relationship problem (nonintimate)	431 (5.9)	80 (3.9)	**511 (5.5)**
Victim of interpersonal violence during past month	89 (1.2)	130 (6.4)	**219 (2.4)**
Jealousy (lovers’ triangle)	151 (2.1)	68 (3.3)	**219 (2.4)**
Perpetrator of interpersonal violence during past month	120 (1.7)	19 (<1.0)	**139 (1.5)**
**Life stressor**			
Argument or conflict	2,343 (32.3)	567 (27.7)	**2,910 (31.3)**
Physical fight (two persons, not a brawl)	1,080 (14.9)	169 (8.3)	**1,249 (13.4)**
Crisis during previous or upcoming 2 weeks	511 (7.0)	257 (12.6)	**768 (8.3)**
History of child abuse/neglect	63 (<1.0)	49 (2.4)	**112 (1.2)**
**Crime and criminal activity**			
Precipitated by another crime	2,131 (29.4)	493 (24.1)	**2,624 (28.2)**
Crime in progress^¶^	1,349 (63.3)	298 (60.4)	**1,647 (62.8)**
Drug involvement	975 (13.4)	109 (5.3)	**1,084 (11.7)**
Gang related	826 (11.4)	74 (3.6)	**900 (9.7)**
Terrorist attack	7 (<1.0)	3 (<1.0)	**10 (<1.0)**
**Homicide event**			
Drive-by shooting	789 (10.9)	74 (3.6)	**863 (9.3)**
Walk-by assault	702 (9.7)	46 (2.2)	**748 (8.0)**
Victim used a weapon	540 (7.4)	28 (1.4)	**568 (6.1)**
Caretaker abuse/neglect led to death	221 (3.0)	182 (8.9)	**403 (4.3)**
Random violence	229 (3.2)	99 (4.8)	**328 (3.5)**
Mentally ill suspect	155 (2.1)	151 (7.4)	**306 (3.3)**
Justifiable self-defense	236 (3.3)	8 (<1.0)	**244 (2.6)**
Brawl	184 (2.5)	19 (<1.0)	**203 (2.2)**
Victim was a bystander	111 (1.5)	71 (3.5)	**182 (2.0)**
Victim was an intervener assisting a crime victim	83 (1.1)	20 (<1.0)	**103 (1.1)**
Prostitution	21 (<1.0)	28 (1.4)	**49 (<1.0)**
Stalking	10 (<1.0)	23 (1.1)	**33 (<1.0)**
Victim was a police officer on duty	23 (<1.0)	4 (<1.0)	**27 (<1.0)**
Mercy killing	4 (<1.0)	11 (<1.0)	**15 (<1.0)**
Hate crime	14 (<1.0)	1 (<1.0)	**15 (<1.0)**
Total**	**7,250 (78.8)**	**2,045 (87.1)**	**9,295 (80.5)**

* Includes homicides with one or more precipitating circumstances. Total numbers do not equal the sums of the columns because more than one circumstance could have been present per decedent.

^†^ Denominator includes those homicides with one or more precipitating circumstances. The sums of percentages in columns exceed 100% because more than one circumstance could have been present per decedent.

^§^ Alaska, Arizona, Colorado, Connecticut, Delaware, Georgia, Illinois, Indiana, Iowa, Kansas, Kentucky, Maine, Maryland, Massachusetts, Michigan, Minnesota, Nevada, New Hampshire, New Jersey, New Mexico, New York, North Carolina, Ohio, Oklahoma, Oregon, Pennsylvania, Rhode Island, South Carolina, Utah, Vermont, Virginia, Washington, West Virginia, and Wisconsin. Illinois, Pennsylvania, and Washington collected data on ≥80% of violent deaths in their state, in accordance with requirements under which these states were funded. Data for California are for violent deaths that occurred in four counties (Los Angeles, Sacramento, Shasta, and Siskiyou).

^¶^ Denominator includes those decedents involved in an incident that was precipitated by another crime.

** Circumstances were unknown for 2,255 (1,953 males and 302 females); total number of homicide decedents = 11,550 (9,203 males and 2,347 females).

Among the identified homicide circumstances, several differences were noted by decedent’s sex, and intimate partner violence accounted for the largest percentage difference (Table 5). Intimate partner violence was a precipitating circumstance for approximately 41.9% of homicides among females but only 7.3% of homicides among males. A larger proportion of female victims than male victims of homicide also resulted from caretaker abuse or neglect (8.9% versus 3.0%, respectively) and were perpetrated by a mentally ill suspect (7.4% versus 2.1%, respectively). A larger proportion of homicides of males than females was preceded by a physical fight (14.9% versus 8.3%, respectively), involved drugs (13.4% versus 5.3%, respectively), and was gang related (11.4% versus 3.6%, respectively). A larger proportion of male victims than female victims also was reported to have used a weapon during the incident (7.4% versus 1.4%, respectively).

### Intimate Partner Violence–Related Homicides

#### Sex, Age Group, and Race/Ethnicity

For 2017, a total of 34 NVDRS states, four California counties, and the District of Columbia collected data on 1,382 homicides that were known to be related to intimate partner violence, which included 526 deaths among males and 856 deaths among females (Table 6). Overall, 22.5% of intimate partner violence–related deaths involved a homicide followed by a suicide, which occurred for a larger percentage of female victims than male victims (32.7% versus 5.9%, respectively). Approximately 9.7% of intimate partner violence–related deaths involved multiple homicide victims in the incident (10.6% of females and 8.2% of males). The largest proportion occurred among adults aged 35–44 years and 45–54 years (22.6% and 19.0%, respectively). Most intimate partner violence–related homicide victims were non-Hispanic Whites (43.2% of males and 56.8% of females) or non-Hispanic Blacks (44.5% of males and 23.9% of females).

**TABLE 6:** Number and percentage* of intimate partner violence–related homicides,^†^ by decedent’s sex, age group, race/ethnicity, method used, location where injury occurred, and victim-suspect relationship — National Violent Death Reporting System, 34 states,^§^ four California counties, and the District of Columbia, 2017

Characteristic	Male	Female	Total
	No. (%)	No. (%)	No. (%)
**Age group (yrs)**			
<1	1 (<1.0)	3 (<1.0)	**4 (<1.0)**
1–4	1 (<1.0)	2 (<1.0)	**3 (<1.0)**
5–9	4 (<1.0)	2 (<1.0)	**6 (<1.0)**
10–14	7 (1.3)	1 (<1.0)	**8 (<1.0)**
15–19	20 (3.8)	27 (3.2)	**47 (3.4)**
20–24	40 (7.6)	83 (9.7)	**123 (8.9)**
25–29	75 (14.3)	106 (12.4)	**181 (13.1)**
30–34	78 (14.8)	126 (14.7)	**204 (14.8)**
35–44	120 (22.8)	193 (22.5)	**313 (22.6)**
45–54	104 (19.8)	159 (18.6)	**263 (19.0)**
55–64	50 (9.5)	74 (8.6)	**124 (9.0)**
65–74	20 (3.8)	45 (5.3)	**65 (4.7)**
75–84	4 (<1.0)	26 (3.0)	**30 (2.2)**
≥85	2 (<1.0)	9 (1.1)	**11 (<1.0)**
**Race/Ethnicity**			
White, non-Hispanic	227 (43.2)	486 (56.8)	**713 (51.6)**
Black, non-Hispanic	234 (44.5)	205 (23.9)	**439 (31.8)**
American Indian/Alaska Native, non-Hispanic	7 (1.3)	15 (1.8)	**22 (1.6)**
Asian/Pacific Islander	11 (2.1)	21 (2.5)	**32 (2.3)**
Hispanic^¶^	45 (8.6)	126 (14.7)	**171 (12.4)**
Other	2 (<1.0)	3 (<1.0)	**5 (<1.0)**
**Method**			
Firearm	361 (68.6)	520 (60.7)	**881 (63.7)**
Sharp instrument	111 (21.1)	146 (17.1)	**257 (18.6)**
Hanging/strangulation/suffocation	11 (2.1)	70 (8.2)	**81 (5.9)**
Blunt instrument	18 (3.4)	54 (6.3)	**72 (5.2)**
Personal weapons (e.g., hands, feet, or fists)	5 (<1.0)	39 (4.6)	**44 (3.2)**
Fire/burns	6 (1.1)	9 (1.1)	**15 (1.1)**
Motor vehicle (e.g., bus, motorcycle, or other transport vehicle)	7 (1.3)	8 (<1.0)	**15 (1.1)**
Other method**	3 (<1.0)	4 (<1.0)	**7 (<1.0)**
Unknown	4 (<1.0)	6 (<1.0)	**10 (<1.0)**
**Location**			
House/apartment	397 (75.5)	680 (79.4)	**1,077 (77.9)**
Street/highway	49 (9.3)	32 (3.7)	**81 (5.9)**
Motor vehicle	13 (2.5)	42 (4.9)	**55 (4.0)**
Other location^††^	57 (10.8)	82 (9.6)	**139 (10.1)**
Unknown	10 (1.9)	20 (2.3)	**30 (2.2)**
**Relationship of victim to suspect^§§^**			
Current intimate partner	200 (41.9)	640 (77.3)	**840 (64.4)**
Former intimate partner	38 (8.0)	123 (14.9)	**161 (12.3)**
Other person, known to victim	108 (22.6)	14 (1.7)	**122 (9.3)**
Acquaintance/friend	56 (11.7)	7 (<1.0)	**63 (4.8)**
Child^¶¶^	13 (2.7)	12 (1.4)	**25 (1.9)**
Intimate partner, unknown whether current or former	4 (<1.0)	18 (2.2)	**22 (1.7)**
Other relative	11 (2.3)	9 (1.1)	**20 (1.5)**
Stranger	15 (3.1)	4 (<1.0)	**19 (1.5)**
Parent^¶¶^	14 (2.9)	1 (<1.0)	**15 (1.1)**
Other relationship***	18 (3.8)	0 (0.0)	**18 (1.4)**
**Total**	**526 (100)**	**856 (100)**	**1,382 (100)**

* Percentages might not total 100% due to rounding.

^†^ Includes victims killed by an intimate partner (e.g., current, former, or unspecified spouse, boyfriend, or girlfriend) and victims killed during an intimate partner violence-related homicide who were not the intimate partner of the suspect (e.g., family, friends, or others who might have intervened in intimate partner violence, such as first responders or bystanders).

^§^ Alaska, Arizona, Colorado, Connecticut, Delaware, Georgia, Illinois, Indiana, Iowa, Kansas, Kentucky, Maine, Maryland, Massachusetts, Michigan, Minnesota, Nevada, New Hampshire, New Jersey, New Mexico, New York, North Carolina, Ohio, Oklahoma, Oregon, Pennsylvania, Rhode Island, South Carolina, Utah, Vermont, Virginia, Washington, West Virginia, and Wisconsin. Illinois, Pennsylvania, and Washington collected data on ≥80% of violent deaths in their state, in accordance with requirements under which these states were funded. Data for California are for violent deaths that occurred in four counties (Los Angeles, Sacramento, Shasta, and Siskiyou).

^¶^ Includes persons of any race.

** Other method includes (in descending order) fall, poisoning, and intentional neglect.

^††^ Other location includes (in descending order) parking lot/public garage/public transport, hotel/motel, commercial/retail area, natural area, park/playground/sports or athletic area, hospital or medical facility, bar/nightclub, farm, office building, abandoned house/building/warehouse, industrial or construction area, jail/prison, and other unspecified location.

^§§^ Percentage is based on the number of intimate partner violence–related homicide decedents with a known victim-suspect relationship (n = 1,305; 477 males and 828 females).

^¶¶^ Includes adoptive family members (e.g., adopted child), stepfamily members (e.g., stepparent), and foster family members (e.g., foster child).

*** Other relationship includes (in descending order) intimate partner of suspect's parent (e.g., teenager kills his mother’s boyfriend), child of suspect's boyfriend/girlfriend (e.g., child killed by mother’s boyfriend), and victim was law enforcement officer injured in line of duty.

#### Method, Location of Injury, and Victim-Suspect Relationship

The most common method of injury in intimate partner violence–related homicide was a firearm (63.7%), followed by sharp instrument (18.6%), hanging/strangulation/suffocation (5.9%), and blunt instrument (5.2) (Table 6). A firearm was used in a larger proportion of intimate partner violence–related homicides among males than females (68.6% versus 60.7%, respectively); however, a larger proportion of females died by hanging/strangulation/suffocation than did males (8.2% versus 2.1%, respectively). The majority (77.9%) of intimate partner violence–related homicides occurred in a house/apartment, followed by a street/highway (5.9%) and a motor vehicle (4.0%); however, a larger percentage occurred on a street/highway for males than for females (9.3% versus 3.7%, respectively).

Among intimate partner violence–related homicides, the sex of the suspect was known for most (97.1%) victims (96.4% of males and 97.6% of females). When the sex of the suspect was known, the majority (98.1%) of female victims were killed by a male suspect, whereas approximately half (46.7%) of male victims were killed by a female suspect. The suspect was known for 94.4% of intimate partner violence–related homicides (90.7% of males and 96.7% of females) (Table 6). When the relationship of the victim to the suspect was known, among male victims, 41.9% and 8.0% were perpetrated by a current or former intimate partner, respectively, whereas among female victims, 77.3% and 14.9% were perpetrated by a current or former intimate partner, respectively. For a small percentage of cases, the suspect was an intimate partner but the investigative reports did not specify whether the suspect was a current or former intimate partner (<1.0% of male victims and 2.2% of female victims). A larger proportion of males than females was killed by a person they knew (i.e., other person known to the victim but the exact nature of the relationship was unclear) (22.6% versus 1.7%, respectively) or an acquaintance/friend (11.7% versus <1.0%, respectively).

#### Precipitating Circumstances

Among intimate partner violence–related homicides, several differences were noted in interpersonal conflicts by the victim’s sex (Table 7). Jealousy was a precipitating circumstance in 28.1% of intimate partner violence–related homicides among males compared with 7.9% of females. Among victims of intimate partner violence–related homicide, one in 10 (10.6%) females was known to be a victim of interpersonal violence during the past month compared with 3.4% of males, whereas a larger proportion of males than females was known to have perpetrated interpersonal violence during the past month (8.7% versus 1.2%, respectively). Other nonintimate relationship problems also occurred among a larger proportion of males than females (7.6% versus 1.5%, respectively, for other relationship problem [e.g., a problem with a friend or an associate] and 5.1% versus 2.6%, respectively, for a family relationship problem). An argument or conflict preceded more than half (58.0%) of intimate partner violence–related homicides among males and 38.8% among females. A physical fight preceded a larger proportion among males than females (23.6% versus 10.0%, respectively); however, a similar percentage was precipitated by a recent or impending crisis among males and females (16.0% and 14.4%, respectively).

**TABLE 7:** Number* and percentage^†^ of intimate partner violence–related homicides,^§^ by decedent’s sex and precipitating circumstances — National Violent Death Reporting System, 34 states,^¶^ four California counties, and the District of Columbia, 2017

Precipitating circumstance	Male	Female	Total
	No. (%)	No. (%)	No. (%)
**Mental health/Substance use**			
Substance use problem (excludes alcohol)	75 (14.3)	86 (10.0)	**161 (11.6)**
Current diagnosed mental health problem	26 (4.9)	88 (10.3)	**114 (8.2)**
History of ever being treated for a mental health problem	21 (4.0)	66 (7.7)	**87 (6.3)**
Alcohol problem	37 (7.0)	34 (4.0)	**71 (5.1)**
Current mental health treatment	15 (2.9)	52 (6.1)	**67 (4.8)**
Current depressed mood	5 (<1.0)	12 (1.4)	**17 (1.2)**
Other addiction (e.g., gambling or sex)	0 (0.0)	1 (<1.0)	**1 (<1.0)**
**Interpersonal**			
Jealousy (lovers’ triangle)	148 (28.1)	68 (7.9)	**216 (15.6)**
Victim of interpersonal violence during past month	18 (3.4)	91 (10.6)	**109 (7.9)**
Perpetrator of interpersonal violence during past month	46 (8.7)	10 (1.2)	**56 (4.1)**
Other relationship problem (nonintimate)	40 (7.6)	13 (1.5)	**53 (3.8)**
Family relationship problem	27 (5.1)	22 (2.6)	**49 (3.5)**
**Life stressor**			
Argument or conflict	305 (58.0)	332 (38.8)	**637 (46.1)**
Physical fight (two persons, not a brawl)	124 (23.6)	86 (10.0)	**210 (15.2)**
Crisis during previous or upcoming 2 weeks	84 (16.0)	123 (14.4)	**207 (15.0)**
History of child abuse/neglect	1 (<1.0)	5 (<1.0)	**6 (<1.0)**
**Crime and criminal activity**			
Precipitated by another crime	132 (25.1)	125 (14.6)	**257 (18.6)**
Crime in progress**	66 (50.0)	62 (49.6)	**128 (49.8)**
Drug involvement	28 (5.3)	16 (1.9)	**44 (3.2)**
Gang related	13 (2.5)	12 (1.4)	**25 (1.8)**
**Homicide event**			
Mentally ill suspect	16 (3.0)	55 (6.4)	**71 (5.1)**
Victim used a weapon	54 (10.3)	14 (1.6)	**68 (4.9)**
Justifiable self-defense	38 (7.2)	4 (<1.0)	**42 (3.0)**
Stalking	7 (1.3)	22 (2.6)	**29 (2.1)**
Caretaker abuse/neglect led to death	6 (1.1)	18 (2.1)	**24 (1.7)**
Victim was an intervener assisting a crime victim	9 (1.7)	8 (<1.0)	**17 (1.2)**
Walk-by assault	11 (2.1)	6 (<1.0)	**17 (1.2)**
Brawl	12 (2.3)	4 (<1.0)	**16 (1.2)**
Drive-by shooting	11 (2.1)	1 (<1.0)	**12 (<1.0)**
Victim was a bystander	3 (<1.0)	5 (<1.0)	**8 (<1.0)**
Mercy killing	0 (0.0)	8 (<1.0)	**8 (<1.0)**
Prostitution	0 (0.0)	6 (<1.0)	**6 (<1.0)**
Random violence	2 (<1.0)	2 (<1.0)	**4 (<1.0)**
**Total**	**526 (100)**	**856 (100)**	**1,382 (100)**

* Includes intimate partner violence–related homicides with one or more precipitating circumstances. Total numbers do not equal the sums of the columns because more than one circumstance could have been present per decedent.

^†^ Denominator includes those intimate partner violence–related homicides with one or more precipitating circumstances. The sums of percentages in columns exceed 100% because more than one circumstance could have been present per decedent.

_§_ Includes victims killed by an intimate partner (e.g., current, former, or unspecified spouse, boyfriend, or girlfriend) and victims killed during an intimate partner violence–related homicide who were not the intimate partner of the suspect (e.g., family, friends, or others who might have intervened in intimate partner violence, such as first responders or bystanders).

^¶^ Alaska, Arizona, Colorado, Connecticut, Delaware, Georgia, Illinois, Indiana, Iowa, Kansas, Kentucky, Maine, Maryland, Massachusetts, Michigan, Minnesota, Nevada, New Hampshire, New Jersey, New Mexico, New York, North Carolina, Ohio, Oklahoma, Oregon, Pennsylvania, Rhode Island, South Carolina, Utah, Vermont, Virginia, Washington, West Virginia, and Wisconsin. Illinois, Pennsylvania, and Washington collected data on ≥80% of violent deaths in their state, in accordance with requirements under which these states were funded. Data for California are for violent deaths that occurred in four counties (Los Angeles, Sacramento, Shasta, and Siskiyou).

** Denominator includes those decedents involved in an incident that was precipitated by another crime.

A larger proportion of intimate partner violence–related homicides among males than among females was precipitated by a crime (25.1% versus 14.6%, respectively), with approximately half of these involving a crime in progress (50.0% among males and 49.6% among females) (Table 7). A larger proportion of intimate partner violence–related homicides of males than of females also involved drugs (5.3% versus 1.9%, respectively). Among victims, males also used a weapon in a larger proportion of intimate partner violence–related homicides than did females (10.3% versus 1.6%, respectively); however, a larger proportion of females than males was killed by a mentally ill suspect (6.4% versus 3.0%, respectively).

### Legal Intervention Deaths

#### Sex, Age Group, and Race/Ethnicity

For 2017, a total of 34 NVDRS states, four California counties, and the District of Columbia collected data on 629 legal intervention death incidents involving 635 deaths. Almost all legal intervention deaths were among males (94.8%) (Table 8). The highest rate of legal intervention death was among men aged 25–29 years (1.5 per 100,000 population), followed by men aged 30–34 years (1.4 per 100,000 population). Non-Hispanic White males accounted for nearly half (44.4%) of all legal intervention deaths; however, non-Hispanic American Indian/Alaska Native males had the highest legal intervention death rate (2.8 per 100,000 population), seven times the rate for non-Hispanic White males (0.4 per 100,000). The legal intervention death rate for non-Hispanic Black males (1.2 per 100,000) was three times the rate for non-Hispanic White males. The legal intervention death rate for Hispanic males was 0.8 per 100,000 population.

**TABLE 8:** Number, percentage,* and rate^†^ of legal intervention^§^ deaths, by decedent’s sex, age group, race/ethnicity, method used, and location where injury occurred — National Violent Death Reporting System, 34 states,^¶^ four California counties, and the District of Columbia, 2017

Characteristic	Male		Female		Total	
	No. (%)	Rate	No. (%)	Rate	No. (%)	Rate
**Age group (yrs)**						
<10	0 (0.0)	—**	0 (0.0)	—	**0 (0.0)**	**—**
10–14	1 (<1.0)	—	2 (6.1)	—	**3 (<1.0)**	**—**
15–19	35 (5.8)	0.5	2 (6.1)	—	**37 (5.8)**	**0.3**
20–24	73 (12.1)	1.0	4 (12.1)	—	**77 (12.1)**	**0.6**
25–29	112 (18.6)	1.5	5 (15.2)	—	**117 (18.4)**	**0.8**
30–34	94 (15.6)	1.4	7 (21.2)	—	**101 (15.9)**	**0.7**
35–44	133 (22.1)	1.0	6 (18.2)	—	**139 (21.9)**	**0.5**
45–54	97 (16.1)	0.7	5 (15.2)	—	**102 (16.1)**	**0.4**
55–64	39 (6.5)	0.3	1 (3.0)	—	**40 (6.3)**	**0.2**
65–74	14 (2.3)	—	1 (3.0)	—	**15 (2.4)**	**—**
75–84	3 (<1.0)	—	0 (0.0)	—	**3 (<1.0)**	**—**
≥85	1 (<1.0)	—	0 (0.0)	—	**1 (<1.0)**	**—**
Unknown	0 (0.0)	—	0 (0.0)	—	**0 (0.0)**	**—**
**Race/Ethnicity**						
White, non-Hispanic	282 (46.8)	0.4	23 (69.7)	<0.1	**305 (48.0)**	**0.2**
Black, non-Hispanic	159 (26.4)	1.2	2 (6.1)	—	**161 (25.4)**	**0.6**
American Indian/Alaska Native, non-Hispanic	27 (4.5)	2.8	2 (6.1)	—	**29 (4.6)**	**1.5**
Asian/Pacific Islander	11 (1.8)	—	1 (3.0)	—	**12 (1.9)**	**—**
Hispanic^††^	123 (20.4)	0.8	5 (15.2)	—	**128 (20.2)**	**0.4**
Other	0 (0.0)	—	0 (0.0)	—	**0 (0.0)**	**—**
Unknown	0 (0.0)	—	0 (0.0)	—	**0 (0.0)**	**—**
**Method**						
Firearm	561 (93.2)	0.6	25 (75.8)	<0.1	**586 (92.3)**	**0.3**
Motor vehicle (e.g., bus, motorcycle, or other transport vehicle)	19 (3.2)	—	6 (18.2)	—	**25 (3.9)**	**<0.1**
Personal weapons (e.g., hands, feet, or fists)	7 (1.2)	—	0 (0.0)	—	**7 (1.1)**	**—**
Blunt instrument	2 (<1.0)	—	2 (6.1)	—	**4 (<1.0)**	**—**
Hanging/strangulation/suffocation	2 (<1.0)	—	0 (0.0)	—	**2 (<1.0)**	**—**
Poisoning	2 (<1.0)	—	0 (0.0)	—	**2 (<1.0)**	**—**
Fire/burns	1 (<1.0)	—	0 (0.0)	—	**1 (<1.0)**	**—**
Drowning	0 (0.0)	—	0 (0.0)	—	**0 (0.0)**	**—**
Fall	0 (0.0)	—	0 (0.0)	—	**0 (0.0)**	**—**
Intentional neglect	0 (0.0)	—	0 (0.0)	—	**0 (0.0)**	**—**
Sharp instrument	0 (0.0)	—	0 (0.0)	—	**0 (0.0)**	**—**
Other (single method)	6 (<1.0)	—	0 (0.0)	—	**6 (<1.0)**	**—**
Unknown	2 (<1.0)	—	0 (0.0)	—	**2 (<1.0)**	**—**
**Location**						
House/apartment	235 (39.0)	0.2	12 (36.4)	—	**247 (38.9)**	**0.1**
Street/highway	147 (24.4)	0.2	11 (33.3)	—	**158 (24.9)**	**<0.1**
Motor vehicle	81 (13.5)	<0.1	8 (24.2)	—	**89 (14.0)**	**<0.1**
Parking lot/public garage/public transport	39 (6.5)	<0.1	1 (3.0)	—	**40 (6.3)**	**<0.1**
Commercial/retail area	25 (4.2)	<0.1	0 (0.0)	—	**25 (3.9)**	**<0.1**
Natural area	13 (2.2)	—	1 (3.0)	—	**14 (2.2)**	**—**
Other location^§§^	52 (8.6)	—	0 (0.0)	—	**52 (8.2)**	**—**
Unknown	10 (1.7)	—	0 (0.0)	—	**10 (1.6)**	**—**
**Total**	**602 (100)**	**0.6**	**33 (100)**	**<0.1**	**635 (100)**	**0.3**

* Percentages might not total 100% due to rounding.

^†^ Per 100,000 population.

^§^ The term “legal intervention” does not denote the lawfulness or legality of the circumstances surrounding the death.

^¶^ Alaska, Arizona, Colorado, Connecticut, Delaware, Georgia, Illinois, Indiana, Iowa, Kansas, Kentucky, Maine, Maryland, Massachusetts, Michigan, Minnesota, Nevada, New Hampshire, New Jersey, New Mexico, New York, North Carolina, Ohio, Oklahoma, Oregon, Pennsylvania, Rhode Island, South Carolina, Utah, Vermont, Virginia, Washington, West Virginia, and Wisconsin. Illinois, Pennsylvania, and Washington collected data on ≥80% of violent deaths in their state, in accordance with requirements under which these states were funded. Data for California are for violent deaths that occurred in four counties (Los Angeles, Sacramento, Shasta, and Siskiyou). Denominators for the rates for these four states (California, Illinois, Pennsylvania, and Washington) represent only the populations of the counties from which the data were collected.

** Rates are not reported when number of decedents is <20 or when characteristic response is “other” or “unknown.”

^††^ Includes persons of any race.

^§§^ Other location includes (in descending order) hotel/motel, office building, jail/prison, park/playground/sports or athletic area, hospital or medical facility, bar/nightclub, industrial or construction area, railroad tracks, synagogue/church/temple, abandoned house/building/warehouse, bridge, cemetery/graveyard/other burial ground, farm, preschool/school/college/school bus, and other unspecified location.

#### Method and Location of Injury

A firearm was used in the majority (92.3%) of legal intervention deaths (Table 8). Legal intervention deaths occurred most frequently in a house/apartment (38.9%), followed by a street/highway (24.9%) and a motor vehicle (14.0%).

#### Precipitating Circumstances

Precipitating circumstances were identified in 98.6% of legal intervention deaths (Table 9). When a specific type of crime was known to have precipitated a legal intervention death (n = 569), the type of crime was most frequently assault/homicide (56.8%), followed by robbery (10.2%), motor vehicle theft (9.5%), burglary (5.4%), and drug trade (1.9%) (Supplementary Table S13, https://stacks.cdc.gov/view/cdc/96308). In approximately two thirds (69.2%) of legal intervention deaths that were precipitated by another crime, a serious crime was reportedly in progress at the time of the incident (Table 9); in the remaining cases, a serious crime was reported to have occurred before the incident. The decedent reportedly used a weapon in 70.9% of cases. In 27.3% of legal intervention deaths with known circumstances, a substance use problem (other than alcohol) was reported as a contributing factor, and 19.6% of decedents reportedly had a current diagnosed mental health problem. A recent or impending crisis during the previous or upcoming 2 weeks was cited in 14.4% and an argument or conflict in 14.5% of legal intervention deaths. Among legal intervention deaths with known circumstances, being a perpetrator of interpersonal violence during the past month (9.6%), intimate partner violence (8.8%), drug involvement (6.9%), and family relationship problems (5.1%) were other notable precipitating circumstances.

**TABLE 9:** Number* and percentage^†^ of legal intervention^§^ deaths, by decedent’s sex and precipitating circumstances — National Violent Death Reporting System, 34 states,^¶^ four California counties, and the District of Columbia, 2017

Precipitating circumstance	Male	Female	Total
	No. (%)	No. (%)	No. (%)
**Mental health/Substance use**			
Substance use problem (excludes alcohol)	160 (27.0)	11 (33.3)	**171 (27.3)**
Current diagnosed mental health problem	117 (19.7)	6 (18.2)	**123 (19.6)**
History of ever being treated for a mental health problem	74 (12.5)	8 (24.2)	**82 (13.1)**
Alcohol problem	58 (9.8)	4 (12.1)	**62 (9.9)**
Current mental health treatment	41 (6.9)	4 (12.1)	**45 (7.2)**
Current depressed mood	30 (5.1)	2 (6.1)	**32 (5.1)**
Other addiction (e.g., gambling or sex)	1 (<1.0)	0 (0.0)	**1 (<1.0)**
**Interpersonal**			
Perpetrator of interpersonal violence during past month	60 (10.1)	0 (0.0)	**60 (9.6)**
Intimate partner violence related	52 (8.8)	3 (9.1)	**55 (8.8)**
Family relationship problem	32 (5.4)	0 (0.0)	**32 (5.1)**
Other relationship problem (nonintimate)	19 (3.2)	0 (0.0)	**19 (3.0)**
Jealousy (lovers’ triangle)	3 (<1.0)	0 (0.0)	**3 (<1.0)**
Victim of interpersonal violence during past month	1 (<1.0)	0 (0.0)	**1 (<1.0)**
**Life stressor**			
Argument or conflict	88 (14.8)	3 (9.1)	**91 (14.5)**
Crisis during previous or upcoming 2 weeks	88 (14.8)	2 (6.1)	**90 (14.4)**
Physical fight (two persons, not a brawl)	55 (9.3)	2 (6.1)	**57 (9.1)**
History of child abuse/neglect	0 (0.0)	0 (0.0)	**0 (0.0)**
**Crime and criminal activity**			
Crime in progress**	376 (69.8)	18 (60.0)	**394 (69.2)**
Drug involvement	38 (6.4)	5 (15.2)	**43 (6.9)**
Gang related	12 (2.0)	1 (3.0)	**13 (2.1)**
Terrorist attack	0 (0.0)	0 (0.0)	**0 (0.0)**
**Legal intervention event**			
Victim used a weapon	424 (71.5)	20 (60.6)	**444 (70.9)**
Brawl	9 (1.5)	0 (0.0)	**9 (1.4)**
Victim was a bystander	3 (<1.0)	1 (3.0)	**4 (<1.0)**
Stalking	4 (<1.0)	0 (0.0)	**4 (<1.0)**
Random violence	3 (<1.0)	0 (0.0)	**3 (<1.0)**
Victim was an intervener assisting a crime victim	1 (<1.0)	0 (0.0)	**1 (<1.0)**
Victim was a police officer on duty	0 (0.0)	0 (0.0)	**0 (0.0)**
Mentally ill suspect	0 (0.0)	0 (0.0)	**0 (0.0)**
Prostitution	0 (0.0)	0 (0.0)	**0 (0.0)**
**Total^††^**	**593 (98.5)**	**33 (100)**	**626 (98.6)**

* Includes deaths with one or more precipitating circumstances. Total numbers do not equal the sums of the columns because more than one circumstance could have been present per decedent.

^†^ Denominator includes those deaths with one or more precipitating circumstances. The sums of percentages in columns exceed 100% because more than one circumstance could have been present per decedent.

^§^ The term legal intervention does not denote the lawfulness or legality of the circumstances surrounding the death.

^¶^ Alaska, Arizona, Colorado, Connecticut, Delaware, Georgia, Illinois, Indiana, Iowa, Kansas, Kentucky, Maine, Maryland, Massachusetts, Michigan, Minnesota, Nevada, New Hampshire, New Jersey, New Mexico, New York, North Carolina, Ohio, Oklahoma, Oregon, Pennsylvania, Rhode Island, South Carolina, Utah, Vermont, Virginia, Washington, West Virginia, and Wisconsin. Illinois, Pennsylvania, and Washington collected data on ≥80% of violent deaths in their state, in accordance with requirements under which these states were funded. Data for California are for violent deaths that occurred in four counties (Los Angeles, Sacramento, Shasta, and Siskiyou).

** Denominator includes those decedents involved in an incident that was precipitated by another crime (n = 569; 539 males and 30 females).

^††^ Circumstances were unknown for nine decedents (all males); total number of legal intervention deaths = 635 (602 males and 33 females).

### Unintentional Firearm Deaths

#### Sex, Age Group, and Race/Ethnicity

In 2017, a total of 34 NVDRS states, four California counties, and the District of Columbia collected data on 253 incidents involving 254 unintentional firearm deaths. Approximately half (n = 128; 50.4%;) of these deaths were self-inflicted, and 92 deaths (36.2%) were known to be inflicted by another person; for the remaining 34 deaths (13.4%), the person who inflicted the injury was not known. Males accounted for 82.7% of decedents (Table 10). Persons aged ≤24 years accounted for approximately half (51.2%) of all unintentional firearm deaths. Approximately 18.1% of decedents were aged <15 years. The majority of decedents were non-Hispanic Whites (56.3%), followed by non-Hispanic Blacks (27.2%).

**TABLE 10:** Number and percentage* of unintentional firearm deaths, by decedent’s sex, age group, race/ethnicity, location where injury occurred, and type of firearm — National Violent Death Reporting System, 34 states,^†^ four California counties, and the District of Columbia, 2017

Characteristic	No. (%)
**Sex**	
Male	210 (82.7)
Female	44 (17.3)
**Age group (yrs)**	
<1	0 (0.0)
1–4	22 (8.7)
5–9	11 (4.3)
10–14	13 (5.1)
15–19	48 (18.9)
20–24	36 (14.2)
25–29	22 (8.7)
30–34	18 (7.1)
35–44	20 (7.9)
45–54	20 (7.9)
55–64	20 (7.9)
65–74	11 (4.3)
75–84	12 (4.7)
≥85	1 (<1.0)
**Race/Ethnicity**	
White, non-Hispanic	143 (56.3)
Black, non-Hispanic	69 (27.2)
American Indian/Alaska Native, non-Hispanic	9 (3.5)
Asian/Pacific Islander	4 (1.6)
Hispanic^§^	29 (11.4)
**Location**	
House/apartment	179 (70.5)
Motor vehicle	22 (8.7)
Natural area	21 (8.3)
Parking lot/public garage/public transport	5 (2.0)
Street/highway	5 (2.0)
Other location^¶^	14 (5.5)
Unknown	8 (3.1)
**Firearm type**	
Handgun	170 (66.9)
Shotgun	24 (9.4)
Rifle	21 (8.3)
Other firearm	2 (<1.0)
Unknown	37 (14.6)
**Total**	**254 (100)**

* Percentages might not total 100% due to rounding.

^†^ Alaska, Arizona, Colorado, Connecticut, Delaware, Georgia, Illinois, Indiana, Iowa, Kansas, Kentucky, Maine, Maryland, Massachusetts, Michigan, Minnesota, Nevada, New Hampshire, New Jersey, New Mexico, New York, North Carolina, Ohio, Oklahoma, Oregon, Pennsylvania, Rhode Island, South Carolina, Utah, Vermont, Virginia, Washington, West Virginia, and Wisconsin. Illinois, Pennsylvania, and Washington collected data on ≥80% of violent deaths in their state, in accordance with requirements under which these states were funded. Data for California are for violent deaths that occurred in four counties (Los Angeles, Sacramento, Shasta, and Siskiyou).

^§^ Includes persons of any race.

^¶^ Other location includes (in descending order) bar/nightclub, commercial/retail area, park/playground/sports or athletic area, synagogue/church/temple, farm, hotel/motel, and other unspecified location.

#### Location of Injury and Firearm Type

Among unintentional firearm deaths, 70.5% occurred in a house/apartment, followed by a motor vehicle (8.7%) and a natural area (8.3%) (Table 10). The majority of unintentional firearm deaths involved a handgun (66.9%), followed by a shotgun (9.4%) and a rifle (8.3%). In 14.6% of unintentional firearm deaths, the firearm type was unknown.

#### Context and Circumstances of Injury

The context and circumstances of injury were identified in 91.3% of unintentional firearm deaths (Table 11). Overall, the most common context of injury was playing with a gun (36.2%), followed by showing the gun to others (10.3%), cleaning the gun (9.1%), and hunting (7.3%). Regarding the circumstances of injury, one fourth (25.0%) of unintentional firearm deaths were caused by a person unintentionally pulling the trigger, 15.1% mistakenly thinking the gun was unloaded, and 7.3% mistakenly thinking the magazine was disengaged.

**TABLE 11:** Number and percentage* of unintentional firearm deaths, by contexts and circumstances of injury — National Violent Death Reporting System, 34 states,^†^ four California counties, and the District of Columbia, 2017

Characteristic	No. (%)
**Context of injury**	
Playing with gun	84 (36.2)
Showing gun to others	24 (10.3)
Cleaning gun	21 (9.1)
Hunting	17 (7.3)
Loading/unloading gun	14 (6.0)
Target shooting	13 (5.6)
Celebratory firing	0 (0.0)
Other context of injury	63 (27.2)
**Circumstance of injury**	
Unintentionally pulled trigger	58 (25.0)
Thought gun was unloaded	35 (15.1)
Thought unloaded, magazine disengaged	17 (7.3)
Gun was dropped	12 (5.2)
Gun fired due to defect or malfunction	8 (3.4)
Thought gun safety was engaged	7 (3.0)
Bullet ricocheted	3 (1.3)
Gun fired while holstering	3 (1.3)
Gun was mistaken for a toy	3 (1.3)
Gun fired while handling safety lock	2 (<1.0)
Other mechanism of injury	52 (22.4)
**Total^§^**	**232 (91.3)**

* Percentages might exceed 100% because one or more circumstances could have been known per death. Number and percentage are reported when the number of deaths is <5 because no particular circumstance identifies a single death.

^†^ Alaska, Arizona, Colorado, Connecticut, Delaware, Georgia, Illinois, Indiana, Iowa, Kansas, Kentucky, Maine, Maryland, Massachusetts, Michigan, Minnesota, Nevada, New Hampshire, New Jersey, New Mexico, New York, North Carolina, Ohio, Oklahoma, Oregon, Pennsylvania, Rhode Island, South Carolina, Utah, Vermont, Virginia, Washington, West Virginia, and Wisconsin. Illinois, Pennsylvania, and Washington collected data on ≥80% of violent deaths in their state, in accordance with requirements under which these states were funded. Data for California are for violent deaths that occurred in four counties (Los Angeles, Sacramento, Shasta, and Siskiyou).

^§^ Circumstances were unknown for 22 decedents; total number of unintentional firearm decedents = 254.

### Deaths of Undetermined Intent

#### Sex, Age Group, and Race/Ethnicity

In 2017, a total of 34 NVDRS states, four California counties, and the District of Columbia collected data on 4,457 incidents involving 4,496 deaths of undetermined intent (Supplementary Table S1, https://stacks.cdc.gov/view/cdc/96308). The overall rate of deaths of undetermined intent was 2.2 per 100,000 population. The rate of deaths of undetermined intent was higher among males than among females (2.9 and 1.5 per 100,000 population, respectively) (Supplementary Table S4, https://stacks.cdc.gov/view/cdc/96308). Over half (58.3%) of deaths of undetermined intent were among adults aged 35–64 years. The rate of deaths of undetermined intent was highest among adults aged 30–34 years (3.8 per 100,000 population), followed by adults aged 45–54 years (3.5 per 100,000 population), 35–44 years (3.4 per 100,000 population), and 25–29 years (3.1 per 100,000 population). Non-Hispanic Whites accounted for the majority (71.1%) of deaths of undetermined intent, whereas non-Hispanic American Indians/Alaska Natives had the highest rate (3.5 per 100,000 population). Among males, non-Hispanic Blacks and non-Hispanic American Indians/Alaska Natives had the highest rate of deaths of undetermined intent (both 4.9 per 100,000 population). Among females, non-Hispanic American Indians/Alaska Natives had the highest rate of deaths of undetermined intent (2.3 per 100,000 population).

#### Method and Location of Injury

Poisoning was the most common method of injury in deaths of undetermined intent (71.0%) (Supplementary Table S4, https://stacks.cdc.gov/view/cdc/96308). No other known method accounted for >4.0% overall. The majority of deaths of undetermined intent occurred in a house/apartment (67.3%), followed by a natural area (5.1%) and a street/highway (3.8%).

#### Toxicology Results of Decedent

Tests for antidepressants, benzodiazepines, and opioids were conducted for 35.9%, 41.1%, and 74.2% of decedents, respectively (Supplementary Table S5, https://stacks.cdc.gov/view/cdc/96308). Results for antidepressants and benzodiazepines were positive in 58.1% and 49.1% of decedents tested for those substances, respectively. Results for opioids (including illicit and prescription) were positive in 78.6% of decedents tested.

#### Precipitating Circumstances

Circumstances were identified in 83.2% of deaths of undetermined intent (Supplementary Table S6, https://stacks.cdc.gov/view/cdc/96308). Among deaths of undetermined intent with known circumstances, 37.2% of decedents had a current diagnosed mental health problem; depression/dysthymia (56.8%), anxiety disorder (23.2%), and bipolar disorder (20.9%) were the most common diagnoses. Among deaths of undetermined intent, 8.0% of decedents had a current depressed mood and 22.5% were receiving mental health treatment at the time of death. Substance use problems (other than alcohol) (69.0%) and alcohol problems (27.2%) were the most commonly reported circumstances. Physical health problems (14.2%) and a recent or impending crisis during the preceding or upcoming 2 weeks (10.2%) were other life stressors identified in deaths of undetermined intent. Among decedents, 9.7% had a history of suicidal thoughts or plans, 8.8% had a history of suicide attempts, and 5.2% had disclosed intent to die by suicide.

### Violent Deaths in Puerto Rico

For 2017, Puerto Rico collected data on 961 incidents involving 1,027 deaths. Homicide (n = 729; 71.0%) accounted for the highest rate of violent death (21.9 per 100,000 population), followed by suicide (n = 276; 26.9%; 9.2 per 100,000 population aged ≥10 years).

#### Homicides

##### Sex, Age Group, and Race/Ethnicity

In 2017, a total of 692 homicides among males and 37 homicides among females were reported in Puerto Rico (Supplementary Table S15, https://stacks.cdc.gov/view/cdc/96308). The overall homicide rate for males was 20.9 times the rate for females (43.8 versus 2.1 per 100,000 population). Among males, the homicide rate was 120.5 per 100,000 population among those aged 18–29 years and 82.4 per 100,000 population among those aged 30–44 years. Most (95.6%) homicide victims were Hispanics.

##### Method, Location of Injury, and Victim-Suspect Relationship

A firearm was used in the majority (90.0%) of homicides (Supplementary Table S15, https://stacks.cdc.gov/view/cdc/96308). A firearm was the most common method used in homicides of both males and females (91.3% and 64.9% respectively); however, the firearm homicide rate for males was 28.6 times the rate for females (40.0 versus 1.4 per 100,000 population, respectively). A street/highway was the most common location (51.9%) of homicide for males, whereas a house/apartment was the most common location (62.2%) of homicide for females.

The victim-suspect relationship was known for 40.5% of homicides (Supplementary Table S15, https://stacks.cdc.gov/view/cdc/96308). When the relationship was known, the suspect for male victims was most often (44.4%) another person known to the victim but the exact nature of the relationship was unclear, whereas the suspect for approximately half (48.1%) of female victims was a current or former intimate partner.

##### Precipitating Circumstances

Precipitating circumstances were identified in 99.6% of homicides (Supplementary Table S16, https://stacks.cdc.gov/view/cdc/96308). Among males, 47.2% of homicides involved illicit drugs and 47.3% were gang related. Approximately one third (33.1%) of homicides among males involved drive-by shootings. Intimate partner violence was identified as a contributing factor in 6.3% of homicides overall; however, a larger proportion of homicides among females was precipitated by intimate partner violence than among males (40.5% versus 4.5%, respectively).

#### Suicides

##### Sex, Age Group, and Race/Ethnicity

In 2017, a total of 276 suicides among persons aged ≥10 years (233 suicides among males and 43 suicides among females) were reported in Puerto Rico (Supplementary Table S17, https://stacks.cdc.gov/view/cdc/96308). The suicide rate for males was 6.1 times the rate for females (16.4 versus 2.7 per 100,000 population aged ≥10 years). The suicide rate among men aged ≥65 years was 25.7 per 100,000 population and among men aged 45–64 years was 22.1 per 100,000 population. The majority (95.7%) of suicide decedents were Hispanics.

##### Method and Location of Injury

Hanging/strangulation/suffocation was the most commonly used method for suicide among both males and females (69.1% and 60.5%, respectively) (Supplementary Table S17, https://stacks.cdc.gov/view/cdc/96308). A firearm was used in 13.7% of suicides among males. The most common location where a suicide took place was a house/apartment for both males and females (81.1% and 79.1%, respectively).

##### Toxicology Results of Decedent

Tests for alcohol were conducted for 98.6% of suicide decedents (Supplementary Table S18, https://stacks.cdc.gov/view/cdc/96308). Among those with positive results for alcohol (34.2%), 61.3% had a BAC ≥0.08 g/dL. Tests for cocaine, marijuana, and opioids were conducted for 98.6%, 60.9%, and 96.4% of decedents, respectively. Results for cocaine and marijuana were positive in 17.6% and 11.9% of decedents tested, respectively. Results for opioids (including illicit and prescription) were positive in 6.8% of decedents tested for these substances.

##### Precipitating Circumstances

Circumstances were identified in 99.6% of suicides (Supplementary Table S19, https://stacks.cdc.gov/view/cdc/96308). Overall, a mental health problem was the most common circumstance among suicide decedents, with 63.6% experiencing a depressed mood at the time of death and 40.0% having a current diagnosed mental health problem.

A crisis during the previous or upcoming 2 weeks was reported among 19.8% of males and 30.2% of females, and 21.2% of males were reported to have had a physical health problem. Among other circumstances related to the suicide, 31.3% of decedents had a history of previous suicide attempts, 28.7% had a history of suicidal thoughts or plans, and 22.5% had disclosed suicidal intent to another person.

## Discussion

Violent deaths affect males and females and persons of all ages, races, and ethnicities. NVDRS provides information on specific manners of violent death and can be used to identify characteristics of and disparities in populations particularly affected by fatal violence. NVDRS data also have the capacity to identify cross-cutting risk factors for multiple forms of violence. These details increase the knowledge base about the circumstances associated with violence and can assist public health authorities and their partners in developing and guiding effective, data-informed approaches to violence prevention.

The occurrence of violent death varies greatly across states, districts, and territories (*1*). This report summarizes data on violent deaths that occurred in 2017 in 34 NVDRS states, four California counties, the District of Columbia, and, for the first time, a U.S. territory (Puerto Rico). The 34 states, four California counties, and District of Columbia represented 63.5% of the U.S. population and accounted for 63.1% of violent deaths in the United States in 2017 (*1*,*8*). In 2019, NVDRS expanded data collection nationwide, providing more comprehensive, accessible, and actionable violent death information that can be used to guide the development of evidence-based violence prevention efforts at local, regional, state, and national levels. Expanding NVDRS to a nationwide system also contributes to the national prevention initiative *Healthy People 2020* objectives to increase the number of states that link data on violent deaths from death certificates, coroner or medical examiner reports, and law enforcement reports at state and local levels and to reduce the number of suicides, homicides, and firearm-related deaths (*13*).

Violence is preventable, and reducing violent deaths in communities is possible with evidence-based approaches (*14*). CDC developed technical packages to assist communities in identifying violence prevention approaches that are based on the best available evidence. The five technical packages describe strategies, approaches, and specific programs, practices, and policies with evidence to reduce the risk for suicide, youth violence, child abuse and neglect, intimate partner violence, and sexual violence. Each technical package considers the multifaceted and interactive effects of different levels of the social ecology, including individual, relationship, family, school, and community factors that influence violence-related outcomes. NVDRS gathers ongoing, systematic, and consistent data on violent deaths that can be used by violence prevention experts within their communities to guide planning and implementation and track outcomes of violence prevention strategies and approaches, such as those outlined in the technical packages.

Demographic variations persist in the manner of death from violence-related injuries. Suicides comprised the majority of violent deaths collected in NVDRS and occurred at higher rates among non-Hispanic American Indians/Alaska Natives and non-Hispanic Whites. Suicide rates were highest among males and adults aged 45–64 years and ≥85 years. One third of suicide decedents had a history of suicidal thoughts or plans, and one fourth had disclosed their suicidal intent. Mental health problems were the most commonly identified circumstance. Past suicidal behavior and mental illness are important risk factors for suicide (*15*), and these circumstances are well documented as important risk factors to target in suicide prevention (*15*,*16*). However, less than one third of suicide decedents were known to be receiving treatment at the time of death, pointing to a gap between those receiving treatment and those who would likely benefit from it. Multiple factors contribute to the risk for suicide (*17*), and the findings in this report indicate that intimate partner problems and recent or impending crises also were common precipitating circumstances. Another factor that might contribute to the risk for suicide is access to lethal means (*15*). A firearm was the most common method used in suicides. Lethal means, such as firearms, provide limited opportunity for intervention and have high case-fatality rates (*15*). Creating protective environments by reducing access to lethal means among persons at risk can be an effective strategy to prevent suicide (*15*).

CDC’s suicide prevention technical package recommends the following seven strategies for reducing suicide and suicidal behaviors: 1) strengthen economic supports, 2) strengthen access to and delivery of suicide care, 3) create protective environments, 4) promote connectedness, 5) teach coping and problem-solving skills, 6) identify and support persons at risk, and 7) lessen harms and prevent future risk (*15*). These strategies support the goals and objectives of the National Strategy for Suicide Prevention (NSSP), which is a comprehensive national agenda for suicide prevention (*18*), and the National Action Alliance for Suicide Prevention’s priority to strengthen community-based prevention (*19*). NVDRS is relevant to the NSSP goals of increasing timeliness and usefulness of surveillance systems related to suicide prevention and evaluating outcomes and effectiveness of suicide prevention interventions. The suicide prevention technical package includes examples of specific approaches that communities can implement to advance each strategy. The findings in this report underscore the importance of approaches outlined in the suicide technical package, such as social-emotional learning programs, enhancing parenting skills and family relationships, and treatment for persons at risk for suicide and treatment to prevent reattempts.

One promising suicide prevention program is *Perfect Depression Care*, which was the precursor to *Zero Suicide* (*15*). This program’s aim is to prevent suicide through systemic change in health and behavioral health care systems with a focus on increasing the safety and support of patients and the health care providers who treat them (*15*). Essential components include training a competent workforce, identifying at-risk persons, and providing evidence-based care with continuous, systemwide coordination (*15*). Two states participating in NVDRS (South Carolina and Colorado) are using their VDRS data to support suicide prevention programs through systems change and the *Zero Suicide* framework (*20*–*23*). The South Carolina Department of Mental Health received a grant from the Substance Abuse and Mental Health Services Administration in 2018 to implement the *Zero Suicide* approach in various health care settings as part of the Adult Suicide Prevention Initiative (*20*). For a program evaluation of the *Zero Suicide* initiative, South Carolina VDRS was part of the evaluation committee and provided data on the prevalence of suicides among adults aged >25 years in South Carolina, trends in circumstances associated with these suicide deaths, and potential areas for prevention (*21*,*22*). These data were used to develop an infographic to bring awareness of South Carolina suicide data for programming staff and stakeholders (*22*). Colorado adopted the *Zero Suicide* framework in 2016 with the goal of expanding the hospital-based framework to multiple systems, including the criminal justice system (*23*). Data from the Colorado VDRS were analyzed for this effort and were used to examine mental health treatment among suicide decedents who had a criminal legal problem that contributed to death and those who did not (*23*). The study found an overall lack of mental health treatment among all suicide decedents (*23*). Only 25.9% of suicide decedents with a contributing criminal legal problem and 30.4% of suicide decedents without a criminal legal problem were reported to be receiving mental health treatment (*23*). These findings highlight the need to expand *Zero Suicide* efforts across multiple systems to increase access to continuous and coordinated mental health treatment to reduce suicides.

Infants experienced high homicide rates, reinforcing the need for prioritizing child abuse and neglect prevention and intervention strategies. Child abuse and neglect are associated with immediate physical injuries, emotional and psychological problems, involvement in risky health behaviors, and a host of broader physical health challenges and long-term health consequences (*24*). CDC’s child abuse and neglect prevention technical package identified the following evidence-based strategies and approaches: 1) strengthening economic supports for families, 2) changing social norms to support parents and positive parenting, 3) providing quality care and education early in life, 4) enhancing parenting skills to promote healthy child development, and 5) intervening to lessen harms and prevent future risk (*24*). Child abuse and neglect are preventable, and the specific approaches described in the technical package can help create safe, stable, and nurturing relationships and environments (*25*) to reduce homicides of infants and children as well as nonfatal child maltreatment.

Homicide rates were highest among non-Hispanic Blacks and non-Hispanic American Indians/Alaska Natives. The homicide rate was more than double the suicide rate in Puerto Rico. Racial and ethnic minorities experience inequitable rates of violent injury and homicide, particularly among youths and young adult males (*26*). These inequities are pervasive and persistent and warrant prioritizing race- and ethnicity-related inequities in the risk for violence (*26*). Racial and ethnic minorities are disproportionately exposed to systemic inequities such as residential segregation, concentrated disadvantage, stress from experiencing racism, limited access to the best educational and employment opportunities, and other conditions that increase the risk for experiencing violence (*26*–*28*). Racial and ethnic minority youths often live in communities with concentrated poverty, stressed economies, residential instability and neighborhood disorganization, and low community cohesion and informal controls (*27*,*28*). All these conditions are associated with violence and violence-related injuries (*27*). Violence prevention efforts will achieve greater population-level decreases in violence through the reduction and elimination of systemic inequities and by targeting salient neighborhood and community-level contributors to violence (*29*). CDC’s youth violence prevention technical package outlines several community- and societal-level programs and approaches (*30*), such as Baltimore’s Safe Streets (*31*), Crime Prevention Through Environmental Design (*32*), business improvement districts (*33*,*34*), and policies, such as the Earned Income Tax Credit (EITC) (*35*,*36*). For example, enhancing household financial security through tax credits, such as the EITC, can help families raise their income while incentivizing work or counterbalancing the costs of child rearing and help create home environments that encourage healthy development (*35*,*36*). Evaluations of these programs and policies have confirmed the value in using these types of approaches to reduce the risk for violence and promote protective community environments (*30*). Evidence also suggests that these approaches and other universal policies that focus on general community improvements can have a substantial impact on decreasing the race/ethnicity gap in violence (*27*).

Homicides among males were most often precipitated by an argument or conflict or during the commission of a crime (predominately assault/homicide). In contrast, approximately 40% of homicides among females were related to intimate partner violence, and a current or former intimate partner was identified as the suspect for approximately half of female homicide victims with known suspects. These findings were consistent with another NVDRS report that highlighted the differential impact of intimate partner violence–related homicide among young and racial/ethnic minority women (*37*). Intimate partner violence affects millions of persons in the United States each year (*38*). Estimates from the 2015 National Intimate Partner and Sexual Violence Survey indicate that approximately 80 million persons in the United States have experienced intimate partner violence (i.e., contact sexual violence, physical violence, and stalking by an intimate partner) at some point in their lives, and approximately 12 million in the previous 12 months (*38*).

States participating in NVDRS have used their VDRS data to examine intimate partner violence–related deaths to support prevention efforts (*39*,*40*). Data from the North Carolina VDRS were used to examine intimate partner homicides that occurred in North Carolina during 2011–2015 (*39*). North Carolina VDRS found that 48.2% of all female homicide victims in North Carolina were victims of intimate partner homicide (versus 5.4% of male homicide victims) (*39*). Another study using data from the Kentucky VDRS examined the role of intimate partner violence among suicides that occurred during 2005–2015 (*40*). Kentucky VDRS determined that 26% of suicide decedents were experiencing problems with a current or former intimate partner (e.g., divorce, jealousy, conflict, or intimate partner violence) that appear to have contributed to their death; of these, intimate partner violence was reported in 43% of cases (*40*). These findings suggest that intimate partner violence might contribute to other manners of violent death, such as suicide, and preventing intimate partner violence might help to reduce the overall number of violent deaths.

CDC’s intimate partner violence prevention technical package outlines several strategies for programs and policies to prevent intimate partner violence and to lessen harms (*41*). Strategies and approaches to prevent and reduce intimate partner violence might occur across different levels of the social ecology, such as engaging men and boys as allies (*41*,*42*); disrupting developmental pathways toward intimate partner violence; creating protective school, workplace, and neighborhood environments (*41*); teaching youths about safe and healthy relationships (*41*,*43*); empowering bystanders; and strengthening economic supports to families (*41*). Prevention efforts can help change harmful gender norms that condone violence and the societal conditions that serve to maintain those norms (*41*,*44*).

The Community Preventive Services Task Force recommends strategies and approaches for youths aged 12–24 years that aim to prevent or reduce intimate partner violence and promote healthier relationships between peers and partners (*45*). The task force recommends approaches that provide information about the warning signs for, or consequences of, intimate partner violence and that also teach healthy relationship skills, promote social norms that protect against violence, and create protective environments (*45*). One such program, CDC’s *Dating Matters,* is a multifaceted, comprehensive prevention model to reinforce protective factors and reduce risk factors for teen dating violence through a school-based program for sixth to eighth graders, training for parents and educators, and a youth communications program (*43*). Local health departments also are encouraged to build prevention capacity and to track indicator data and teen dating violence–related policy (*43*). Youths in the eighth grade exposed to *Dating Matters* demonstrated lower scores on teen dating violence perpetration and victimization, on average across time points and cohorts, compared with students receiving an evidence-based standard of care program (*43*).

For persons who might be experiencing intimate partner violence, victim-centered services such as domestic violence shelters, hotlines, crisis intervention and counseling, medical and legal advocacy, and access to community resources might help to improve outcomes for survivors of violence and reduce the long-term negative health consequences of intimate partner violence (*41*). One patient-centered approach to lessening harms that is recommended by the U.S. Preventive Services Task Force is for health care providers to screen all women of childbearing age for intimate partner violence and to refer those who are found to be at risk to intervention services (*46*,*47*). Screening for intimate partner violence should be conducted in a culturally sensitive way to minimize threats to safety.

Intimate partner violence can extend beyond the violence experienced by intimate partners and can include other victims as well (*48*). Approximately 90% of female victims of intimate partner violence–related homicide were killed by a current or former intimate partner, whereas half of male victims were not intimate partners of the suspects but included family members, friends, bystanders, or other persons who intervened during intimate partner violence–related incidents. Bystander programs teach participants how to recognize situations or behaviors that might become violent and safely and effectively intervene to reduce the likelihood of assault (*41*). These programs might be a promising strategy for reducing intimate partner violence witnessed by others. One bystander program implemented with high school students, *Green Dot*, found significant reductions in dating violence perpetration and victimization after 3 years of program implementation (*49*).

A high prevalence of alcohol was observed among suicide decedents tested for substances, especially with BAC ≥0.08 g/dL. Alcohol and substance use are frequent precipitants of suicide and interpersonal violent behavior (*15*). Alcohol use is a robust predictor of suicidal behavior (*50*), victimization (*51*), and interpersonal violence perpetration (*30*,*41*). Intoxication can lead to disinhibition, enhance feelings of hopelessness and depression, and impair judgment, which can lead to impulsive behaviors (*16*). Alcohol use also can reduce awareness and perception of surrounding risks, thus increasing a person’s vulnerability to being victimized (*52*). Poisoning was the most common method of injury for suicides among females and the most common cause of death among deaths of undetermined intent. Results for opioids (illicit or prescription) were positive in one fourth of suicide decedents tested for these substances, and opioids were the most common substances detected in deaths of undetermined intent, with approximately 80% having positive opioid results among those tested. Opioid overdose has been recognized as an epidemic (*53*). CDC published the *Guideline for Prescribing Opioids for Chronic Pain* to help address the opioid overdose epidemic, support safer prescribing practices, and reduce prescription opioid misuse, opioid use disorder, and overdose (*54*). Building on previous CDC programs focused on opioid overdose and injury prevention, CDC also has implemented comprehensive surveillance and prevention activities through *Overdose Data to Action* to support state and local health departments in obtaining data on overdose morbidity and mortality and using the data to guide prevention and response efforts (*55*–*57*). Other important activities to address the opioid overdose epidemic include expanding naloxone availability and access to medication-assisted treatment, enhancing public health and public safety partnerships, and maximizing the ability of health systems to link persons to treatment and harm-reduction services (*55*–*58*). 

NVDRS collects more complete information than other data sources on legal intervention deaths (*59*) and unintentional firearm deaths (*60*). The rate of legal intervention death was highest among non-Hispanic American Indians/Alaska Natives, and the rate among non-Hispanic Black males was three times that of their non-Hispanic White male counterparts, a finding consistent with previous studies (*61*,*62*). More analyses are needed to increase knowledge about the magnitude and circumstances of these deaths and for developing appropriate prevention strategies and monitoring their effectiveness. NVDRS also has been recognized as a reliable source of data on unintentional firearm deaths (*60*) and for its capability to provide details about victims and shooters (*63*). Approximately half of unintentional firearm deaths were self-inflicted; however, approximately one third were known to be inflicted by another person. Most of these deaths occurred while playing with a gun, accidentally pulling the trigger, or thinking the gun was unloaded, which are of concern, particularly among children (*64*); these findings highlight the importance of safe storage practices and education about safe handling of firearms.

## Limitations

The findings in this report are subject to at least eight limitations. First, NVDRS data are available from a limited number of states, districts, and territories and therefore are not nationally representative. In addition, California, Illinois, Pennsylvania, and Washington data were from a subset of counties and are not representative of these states.

Second, the availability, completeness, and timeliness of data depend on partnerships among VDRS programs and local health departments, vital statistics registrars’ offices, coroners and medical examiners, and law enforcement personnel. Data sharing and communication among partners is particularly challenging when states and territories have independent county coroner systems rather than a centralized coroner or medical examiner system, numerous law enforcement jurisdictions, or both. NVDRS incident data might be limited or incomplete for areas in which these data-sharing relations are not fully developed. Partnerships with local vital statistics registrars’ offices usually are more established because they are part of the public health infrastructure. As part of an active surveillance system, VDRS programs work closely with local vital registrars’ offices to identify deaths meeting the NVDRS case definition and to avoid cases being missed or inappropriately included. CDC also monitors case ascertainment and variable completeness through regular technical assistance calls, which include an internal data quality dashboard in the web-based system that is updated in real time. Overall, <2.0% of cases in NVDRS are missing or have unknown values for variables that represent demographic characteristics (e.g., age, sex, and race/ethnicity) and manners of death.

Third, toxicology data are not collected consistently across all states, districts, and territories or for all alcohol and drug categories. In addition, toxicology testing is not conducted for all decedents; thus, the percentages of decedents with positive results for specific substances might be affected by selective testing patterns in coroner or medical examiner offices (*65*).

Fourth, abstractors are limited to the data included in the investigative reports they receive. For example, information regarding intimate partner relationships might not be routinely collected postmortem, particularly for sexual and gender minorities (*66*), which might result in an underestimate of homicides that are precipitated by intimate partner violence. In addition, reports might not fully reflect all information known about an incident, particularly for homicides and legal intervention deaths, when data are less readily available until a full investigation and adjudication are completed.

Fifth, case definitions present challenges when a single death is classified differently in different documents (e.g., unintentional in a law enforcement report, homicide in a coroner or medical examiner report, and undetermined on the death certificate). NVDRS abstractors reconcile these discrepancies using standard NVDRS case definitions and select a single manner of death on the basis of all source documents (*7*).

Sixth, variations in coding occur depending on the abstractor’s level of experience. For this reason, CDC provides extensive abstractor guidance and training, a coding manual to promote standardized data collection (*7*), and data validation checks. VDRS programs also conduct reabstractions of a subset of cases to test consistency in coding and identify training needs of data abstractors.

Seventh, medical and mental health information (e.g., type of condition and whether the decedent was receiving treatment) often are not captured directly from medical records but from coroner or medical examiner reports and the decedent’s family members and friends. Therefore, completeness and accuracy of this information are limited by the knowledge of the informant.

Finally, protective factor data (i.e., characteristics or circumstances that reduce the risk for violent death) are not collected by NVDRS. This is because of the nature of death certificates, coroner or medical examiner reports, and law enforcement reports, which typically contain only circumstances associated with risk factors.

## Conclusion

Public health surveillance is the foundation for public health practice (*67*). Monitoring the prevalence of violence-related fatal injuries, defining priorities, and informing programmatic and violence prevention activities are essential parts of public health surveillance (*67*). In 2018, NVDRS received funding for nationwide expansion. Beginning in 2019, all 50 states, the District of Columbia, and Puerto Rico participated in NVDRS, a move toward achieving the ultimate goal of providing nationally representative data. This expansion makes violent death information available for local communities to develop prevention efforts and allows for the system’s capacity to measure the need for and effects of violence prevention policies, programs, and practices at the national level.

## Footnotes

* The name of the Institute of Medicine was changed to the National Academy of Medicine, effective July 1, 2015.

† To be included in NVDRS, deaths of undetermined intent must have some evidence of the possibility that the intent was purposeful, including use of a weapon or other evidence that force was used to inflict the injury. Most commonly, the coroner or medical examiner is unsure whether the death was a suicide or unintentional.

§ California began collecting data in 2005 but ended data collection in 2009. In 2017, California collected NVDRS data from all three required sources (i.e., death certificates, coroner or medical examiner reports, and law enforcement reports) from four counties (Los Angeles, Sacramento, Shasta, and Siskiyou).

¶ Frequencies and rates of violent deaths included in this report will differ slightly from the frequencies and rates of violent deaths reported by CDC’s Web-based Injury Statistics Query and Reporting System (WISQARS), which excludes nonresident deaths that occur in participating states and the District of Columbia (i.e., occurrent deaths). NVDRS tracks both resident and occurrent violent deaths in the overall data set, and the numbers in this report reflect both. VDRS programs are expected to collect information on violent deaths among their residents, wherever they occur, and fatal violent injuries occurring within their borders irrespective of the decedent’s residence status. If the states of residence and injury occurrence are both participating NVDRS states, the state of injury occurrence is responsible for collecting the information. By making this differentiation of responsibility, duplicate reporting is avoided.

** Drug trade is defined as the buying, selling, or passing of drugs in exchange for goods or money. Drug involvement includes drug use in addition to drug trade.
